# A novel type-II–II heterojunction for photocatalytic degradation of LEV based on the built-in electric field: carrier transfer mechanism and DFT calculation

**DOI:** 10.1038/s41598-024-60250-z

**Published:** 2024-05-09

**Authors:** Jiaquan Li, Peng Tu, Qian Yang, Yanjun Cui, Chenyang Gao, Hui Zhou, Jun Lu, Hongxia Bian

**Affiliations:** 1 College of Science, Gansu Agricultural University, Lanzhou, 730070 People’s Republic of China; 2 School of Materials Science and Engineering, Lanzhou University of Technology, Lanzhou, 730050 People’s Republic of China

**Keywords:** Ag_2_CO_3_/Bi_2_WO_6_, Photocatalysis, Heterojunction, Levofloxacin, Density functional theory, Analytical chemistry, Photocatalysis, Optical materials

## Abstract

Heterojunctions play a crucial role in improving the absorption of visible light and performance of photocatalysts for organic contaminants degradation in water. In this work, a novel type-II–II Ag_2_CO_3_/Bi_2_WO_6_ (AB) heterojunction was synthesized by hydrothermal reaction and in situ-precipitation methods. The mechanisms of charge transfer and carrier separation at the interface of heterojunctions and the influence on the photocatalytic activity were investigated. The degradation of levofloxacin (LEV) under visible light irradiation was employed to evaluate the photocatalytic performance of AB. The results showed that 85.4% LEV was degraded by AB, which was 1.38 and 1.39 times higher than that of Bi_2_WO_6_ and Ag_2_CO_3_, respectively. The work functions of the different crystal planes in the AB heterojunction, which was calculated by density functional theory, are a significant difference. The Fermi energy (*E*_f_) of Ag_2_CO_3_ (− 6.005 eV) is lower than Bi_2_WO_6_ (− 3.659 eV), but the conduction band (CB) is higher. Therefore, using AB heterojunctions as an example, the research explored the mechanism of type-II–II which CB and *E*_f_ of one semiconductor cannot simultaneously surpass those of another material, based on the built-in electric field theory. Through this analysis, a deeper understanding of type-II heterojunctions was achieved, and providing valuable insights into the behavior of this specific heterojunction system.

## Introduction

With the rapid development of modern industry, the issue of environmental pollution is receiving more and more attention, particularly the contamination of water by organic pollutants, such as antibiotic^1,2^. Antibiotics are widely used in the treatment of broad-spectrum bacterial infections in both humans and animals^3^. However, owing to their high metabolism rates, a substantial portion of antibiotics is excreted through faeces^4^. Subsequently, these antibiotics, in various forms and to varying extents, enter into the environment, giving rise to the proliferation of drug-resistant bacteria and drug-resistant genes, thereby causing a significant public health risk to humans^5^. Therefore, there exists an urgent need to adopt an efficient, rapid, and cost-effective method to treat the issue of antibiotics remediation in water.

Photocatalytic technology has attracted the attention of many scholars as a sustainable and efficient technology for the removal of antibiotics from water^6^. The main mechanism of photocatalytic degradation involves that the photocatalyst absorbs photons, leading to the generation electrons (e^−^) and holes (h^+^) on its surface. This e^−^ and h^+^ can induce O_2_ and water molecules in the solution to participate in redox reactions that yield superoxide radicals (·O_2_^−^) and hydroxyl radicals (·OH). ·O_2_^−^, ·OH and h^+^, which have strong redox capacity, directly can engage in the degradation of antibiotics. Through a series of reactions, organic pollutants can be degraded into carbon dioxide, water, or smaller molecular compounds to achieve the purpose of pollutant removal.

Bismuth-based photocatalysts have attracted extensive attention due to their high charge separation efficiency and efficient use of solar light^7^. Due to its unique layered structure, suitable band gap and high photochemical stability, Bi_2_WO_6_ is considered a promising semiconductor photocatalyst for use in the field of photocatalytic degradation^8–11^. However, the photocatalytic performance of Bi_2_WO_6_ is greatly hindered by the high recombination rate of the photogenerated carriers during the transfer process. Various approaches have been explored to enhance the performance of photocatalysts, including ion doping, noble metals-loading, morphology modulation, and heterojunction assembly^12–15^. Notably, the construction of heterojunction has emerged as an effective strategy to solve the problem of rapid carrier recombination while expanding the range of visible light absorption. Feng Wei et al. synthesized BiOI/Bi_2_WO_6_ nanocomposites, which achieved a 99% degradation of methylene blue (MB) under simulated visible light irradiation, representing a 30% improvement over Bi_2_WO_6_^16^. Similarly, Yang Jun et al. successfully prepared Bi_2_WO_6_/BiOCl heterojunctions using a one-step hydrothermal method, and reached a 93.3% degradation rhodamine B, a 33% enhancement compared to Bi_2_WO_6_^17^. These studies demonstrate that the construction of heterojunction structures can significantly enhance the photocatalytic activity of Bi_2_WO_6_.

Ag_2_CO_3_, known for its good visible light absorption and photocatalytic activity, is also a noteworthy photocatalyst that has attracted substantial attention^18^. In order to overcome the shortcomings of photo-corrosion and electron–hole pair recombination, researchers have dedicated considerable efforts to investigating the construction of heterostructures involving Ag_2_CO_3_, such as Ag_2_CO_3_/TiO_2_^19^, Ag_2_CO_3_/Ag/AgBr^20^, WO_3_/Ag_2_CO_3_^21^, Ag_2_CO_3_/SnFe_2_O_4_^22^, Ag_2_CO_3_/CeO_2_^23^, Ag_2_CO_3_/BiOBr/CdS^24^, among others. These studies have paved the way for novel heterostructure designs aimed at enhancing the stability and photocatalytic performance of Ag_2_CO_3_.

**Figure 1:**
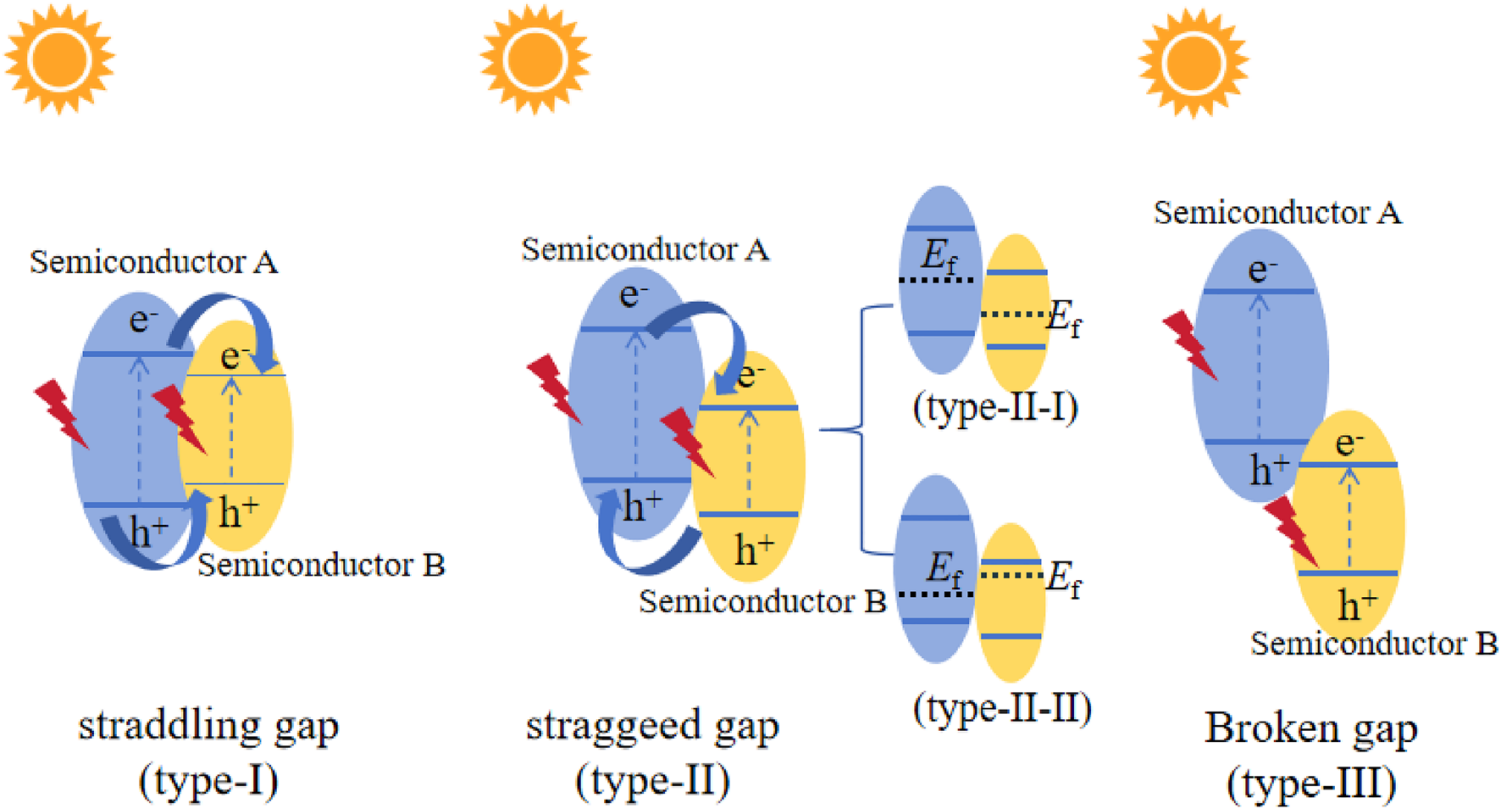
Conventional type-I, II,and III heterojunctions, carrier transfer paths, and classification of type-II.

To elucidate the impact of heterojunctions on photocatalytic activity, scientists have categorized them into three types: straddling (type-I), staggered (type-II), and gap-breaking (type-III), based on the relative positions of the energy bands of the materials^25–27^. About the conventional explanation of type-II heterojunction, e^−^ tends to accumulate in the photocatalyst with a more positive conduction band (CB) potential, while h^+^ transfers to the material with more negative valence band (VB) potential, and enhances light absorption and facilitates charge separation. However, from the kinetic point of view, there is a repulsive force between e^−^ and between h^+^, posing challenges for the migration of charges in the heterojunction theory. In recent years, with the rapid development in the field of photocatalysis, the role of the built-in electric field (IEF) in the transfer of photogenerated carriers has been recognized^28–31^. IEF is mainly determined by the Fermi level (*E*_f_) of the two materials forming the heterojunction. Considering the distribution of the CB and the *E*_f_, the type II heterojunction can be classified into two types: (i) CB and *E*_f_ of one semiconductor are higher than those of another, it is named type-II–I in the article; (ii) CB and *E*_f_ of one semiconductor cannot simultaneously surpass those of another material, it is named type-II–II. Figure 1 is detailed interpretation of the above-mentioned. Regarding the carrier transfer mechanism of type-II–I heterojunction, scholars have offered an explanation based on the IEF theory^32^. However, the photocatalytic mechanism of type-II–II heterojunction remains unexplored. Therefore, we focused on type-II–II heterojunctions and used Ag_2_CO_3_ and Bi_2_WO_6_ to prepare it, where the CB and *E*_f_ of Ag_2_CO_3_ are not higher than those of Bi_2_WO_6_ simultaneously. By investigating the carrier transfer process, we aim to shed light on the influence of the *E*_f_ on the IEF of the heterojunction in the type-II–II heterojunction.

In this work, Ag_2_CO_3_/Bi_2_WO_6_ (AB) nanocomposites were prepared by hydrothermal and in situ precipitation methods. The structure and properties of the materials were characterized and analyzed. The photocatalytic degradation performance of the nanocomposites towards levofloxacin (LEV) was investigated to evaluate their activity, stability, and applicability. The transfer mechanism of photogenerated carriers in AB heterojunctions was explored by density-functional theory (DFT) to improve the existing understanding of type-II heterojunctions and provide valuable insights into the behavior of this specific heterojunction system.

## Materials and methods

### Reagents

Bismuth nitrate pentahydrate (Bi(NO_3_)_3_‧5H_2_O), sodium tungstate dihydrate (Na_2_WO_4_‧2H_2_O), p-benzoquinone (BQ), ethylenediaminetetraacetic acid (EDTA), sodium carbonate (Na_2_CO_3_) and isopropanol (IPA) were purchased from Shanghai Macklin Biochemical Technology Co. Nitric acid and silver nitrate (AgNO_3_) were provided by Sinopharm Chemical Reagent Co. All reagents are analytical grade and do not require further purification. Deionized water (18 μS cm^−1^) was used for all experiments.

### Preparation of photocatalysts

Bi_2_WO_6_ nanosheets were prepared by a hydrothermal method. Initially, 0.97 g of Bi(NO_3_)_3_‧5H_2_O was dispersed in 30 mL of 0.5 mol/L nitric acid solution and stirred for 1 h until completely dissolved. Subsequently, 0.33 g Na_2_WO_4_‧2H_2_O was added into 30 mL of deionized water, completely dissolved, and then added dropwise into Bi(NO_3_)_3_ solution, stirred for 1 h. The resulting suspension was transferred into 80 mL of Teflon-lined stainless autoclave and heated at 160 °C for 18 h. Afterward, the obtained product was washed thrice with deionized water, and the yellow precipitate was dried in an oven at 60 °C for 12 h.

A in situ-precipitation method was used to fabricate AB nanocomposites. Specifically, 2 g of Bi_2_WO_6_ nanoflakes were dispersed in 20 mL of deionized water, and subjected to ultrasonication for 30 min. Subsequently, 14.3 mL of AgNO_3_ solution (0.10 M) was added to the above suspension and stirred for 30 min under dark conditions to promote the adsorption of silver ions on the surface of Bi_2_WO_6_. Then, an equal volume of Na_2_CO_3_ solution (0.05 M) was added and the mixture was stirred for 4 h under dark conditions. The resulting sample was washed three times, filtered, and then dried at 60 °C for 12 h. Once sufficiently dried, the sample was ground and bagged.

In the above AB nanocomposites, the theoretical mass fraction of Ag_2_CO_3_ was 9%, labeled as AB-9. The other nanocomposites (AB-1, AB-3, AB-5, AB-7 and AB-11) with different masses fractions of Ag_2_CO_3_ (1%, 3%, 5%, 7% and 11%) were prepared by adjusting the volumes of AgNO_3_ (1.5 mL, 4.5 mL, 7.5 mL, 10.9 mL, and 18 mL) and Na_2_CO_3_ (1.5 mL, 4.5 mL, 7.5 mL, 10.9 mL, and 18 mL). The synthesis of Ag_2_CO_3_ followed the same way described above, only without the addition of Bi_2_WO_6_.

### Photocatalyst characterization

The crystal structure and surface morphology of the photocatalysts were characterized and analyzed by X-ray powder diffraction (XRD) (China, Puxi General Instrument, XD3) and field emission scanning electron microscopy (SEM) (Germany, ZEISS, Sigma 300). An ultraviolet–visible spectrophotometer (UV–Vis) (Japan, Shimadzu, UV-360Oi Plus) was used to analyze the photo response ability of the samples with a scanning range of 200–800 nm. X-ray photoelectron spectroscopy (XPS) (USA, Thermo Scientific, K-Alpha) was used to examine the surface composition, the valence information, and the surface chemical bonding states of the samples. In addition, the separation efficiency of carriers was evaluated by a fluorescence spectrometer (China, Gangdong Sci &Tech F-380). A 3-electrode electrochemical analysis approach was used for electrochemical impedance spectroscopy (EIS) using an electrochemical workstation (China, Chenhua Instrumen, CHI 660E). The intermediates produced during the process of degradation of LEV were measured and analyzed by liquid chromatography-tandem mass spectrometry (LC–MS) (Japan, Shimadzu, LCMS-8050). The degree of mineralization of LEV was measured by total organic carbon (TOC) (Japan, Shimadzu, TOC-L).

### Photocatalytic degradation experiment

To evaluate the photocatalytic performance of AB nanocomposites, the degradation of LEV was conducted in a temperature-controlled photochemical reactor (NAI-GHY-DSGKW) equipped with multiple tubes. A 500 W xenon lamp (illuminance: 1369 W/m^2^) was employed to simulate the visible light irradiation. Before photocatalytic degradation, 0.05 g of photocatalyst was added into 50 mL solution of LEV (10 mg/L), and stirred under dark conditions for 60 min to reach the adsorption–desorption equilibrium. Then, the light source was turned on to carry out the photocatalytic reaction. At certain time intervals, a 3.0 mL sample was withdrawn from the photoreactor and filtered through a 0.45 µm membrane. The concentration of LEV was determined using UV–vis at a wavelength of 288 nm. To fit the LEV degradation data and calculate the rate constant (*k*) of LEV degradation, a pseudo-primary kinetic model was employed. In addition, IPA, BQ, and EDTA were added as scavengers of ·OH, ·O_2_^−^, and h^+^, respectively, to investigate the contribution of different radicals to the photocatalytic degradation of LEV. The concentration of all three trapping agents were 1.0 mmol/L. ·O_2_^–^ and ·OH were checked by electron spin resonance (ESR) (Germany, Bruker Corporation, Bruker EMX plus,) with 5,5-dimethyl-1-pyrroline N-oxide (DMPO).

### DFT calculations

In this paper, the state density, band structure, and work function of the materials have been calculated using the Materials Studio 2020 (MS) software package based on DFT. The interactions between electrons and ions are described using the projector augmented wave (PAW) method, and the exchange–correlation potential is treated using the PBE generalization in the generalized gradient approximation (GGA). The convergence criteria for the energy and interatomic forces are 10^−5^ eV and 0.01 eV/Å, respectively.

## Results and discussion

### Morphology, structure, and elemental analysis of AB

**Figure 2:**
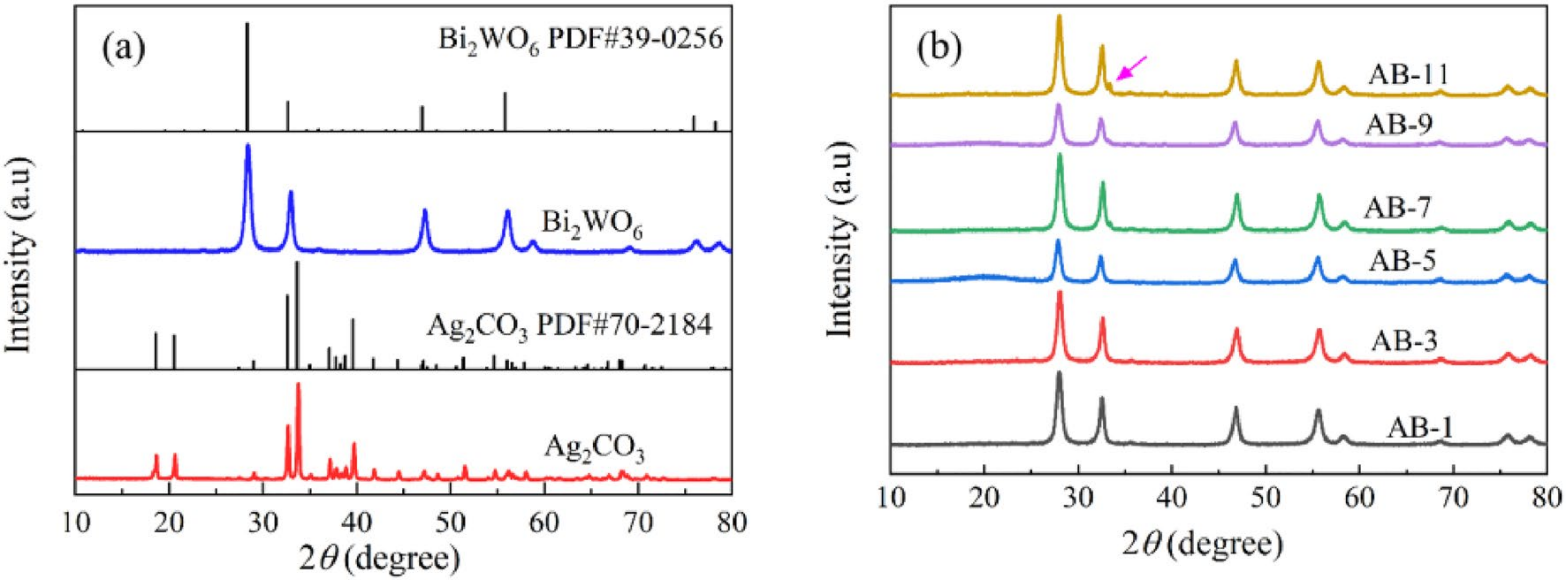
(**a**) XRD patterns of Ag_2_CO_3_ and Bi_2_WO_6_, (**b**) XRD patterns of AB nanocomposites.

The XRD patterns of Ag_2_CO_3_, Bi_2_WO_6_ and AB nanocomposites are shown in Fig. 2. Six diffraction peaks of Ag_2_CO_3_ appear at 18.56°, 20.52°, 32.59°, 33.64°, 37.05°, and 39.57°, corresponding to crystal planes (0 2 0), (1 1 0), (− 1 0 1), (1 3 0), (2 0 0) and (0 3 1), respectively. These results are consistent with the known diffraction pattern of Ag_2_CO_3_ (PDF#70-2184)^33^. The diffraction peaks of Bi_2_WO_6_ (PDF#39-0256) are at 28.3°, 32.67°, 46.9°, and 55.82°, corresponding to (1 3 1), (0 6 0), (2 6 0), and (3 3 1) crystal planes, respectively^34^. Importantly, no diffraction peaks associated with impurities were observed, indicating that the prepared materials have high crystallinity and purity, as shown in Fig. 2a. In Fig. 2b, the XRD pattern of AB nanocomposites reveals primarily the diffraction peaks Bi_2_WO_6_, with the emergence of characteristic peaks of Ag_2_CO_3_ as the Ag_2_CO_3_ content in the material increased. This observation confirms that AB nanocomposites were successfully synthesized.

**Figure 3:**
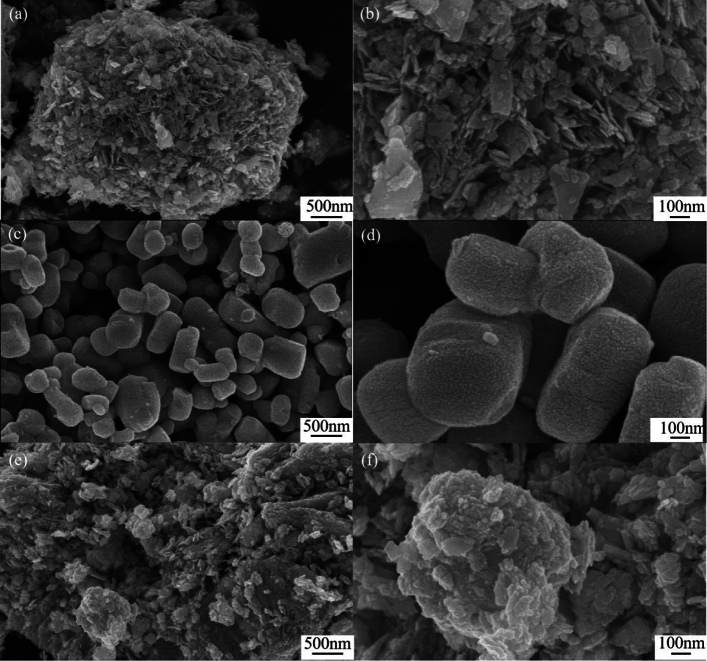
(**a**, **b**) SEM images of Bi_2_WO_6_, (**c**, **d**) SEM images of Ag_2_CO_3_, (**e**, **f**) SEM images of photocatalyst AB-9.

**Figure 4:**
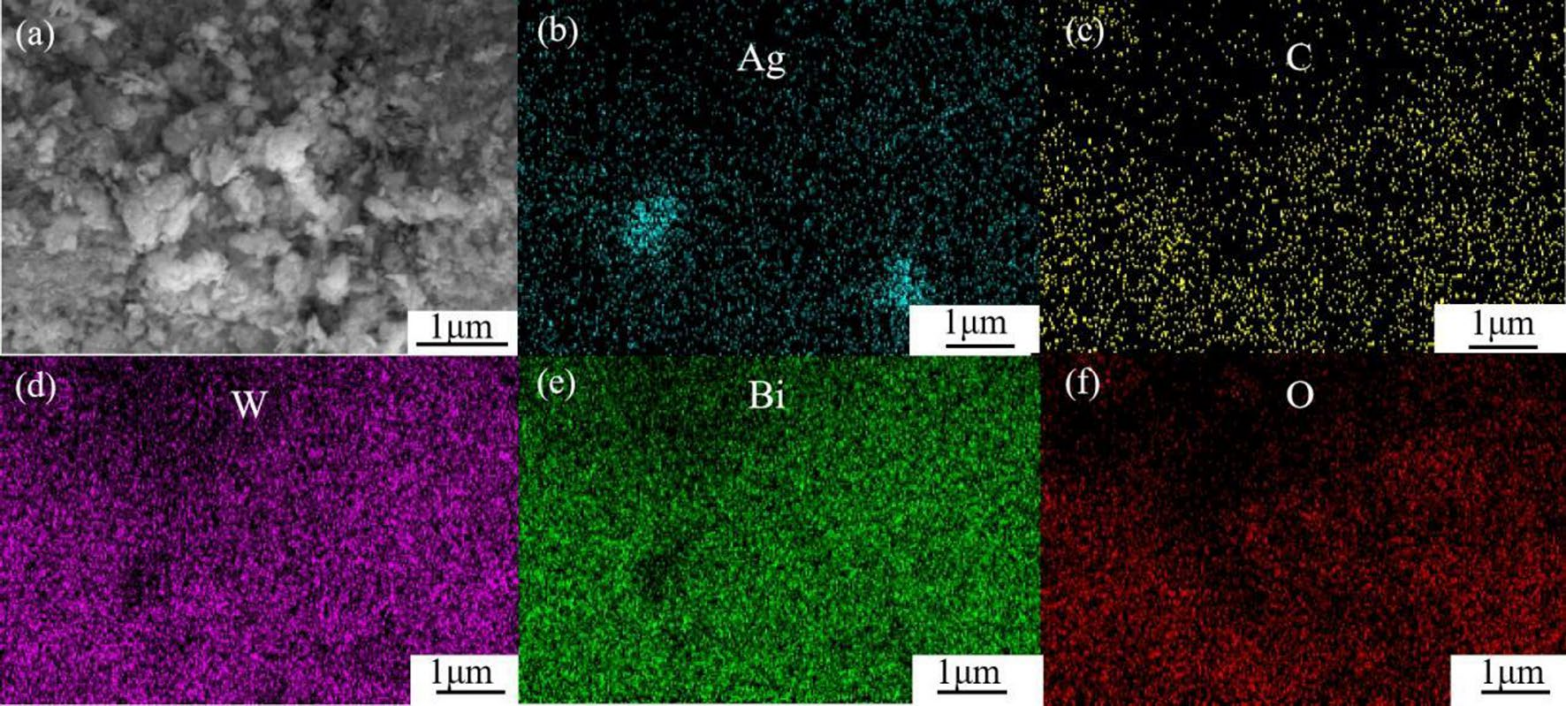
(**a**) Elemental mapping images, (**b**–**f**) Distribution of elements Ag, C, W, Bi, and O in photocatalyst AB-9.

**Figure 5:**
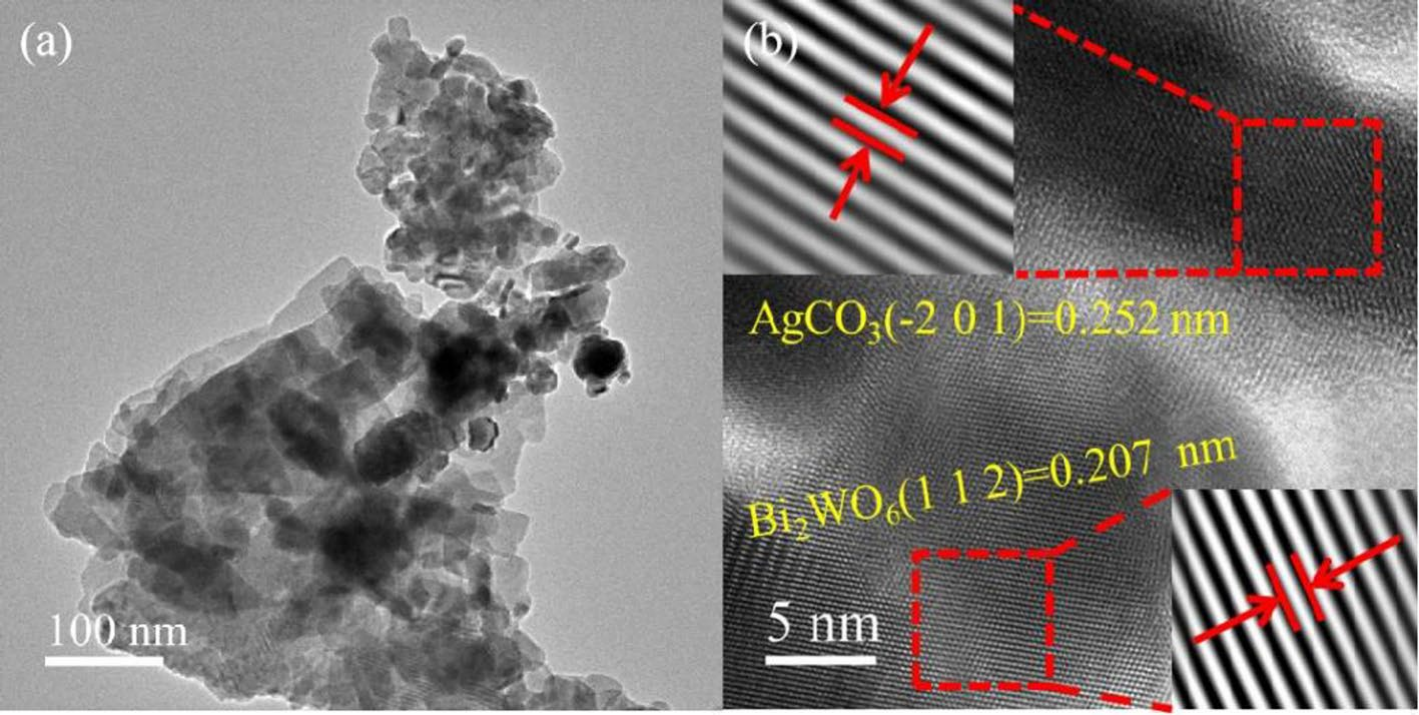
(**a**) TEM images of photocatalyst AB-9, (**b**) HR-TEM images of photocatalyst AB-9.

SEM images of Bi_2_WO_6_, Ag_2_CO_3_, and AB-9 photocatalyst are presented in Fig. 3. Bi_2_WO_6_ exhibits nanosheets with smooth surfaces, forming an overall irregular polyhedral morphology with multiple crevices, as shown in Fig. 3a and b. Ag_2_CO_3_, on the other hand, appears as cuboid and micro-spherical particles with smooth surfaces, as illustrated in Fig. 3c and d. The SEM images of the AB-9 reveal a tight bonding between Ag_2_CO_3_ and Bi_2_WO_6_, forming a new pore structure, as shown in Fig. 3e and f. Figure 4 displays the energy dispersive spectrometer (EDS) spectrum of the AB-9. The presence of Ag, C, W, Bi, and O, is observed in the spectrum. Furthermore, the elements are uniformly distributed throughout the AB-9, indicating successful preparation of the AB nanocomposite. The morphology of AB-9 was further studied by the TEM, as shown in Fig. 5a. TEM images show that the Bi_2_WO_6_ nanosheets are closely covered by Ag_2_CO_3_, which confirms the growth of Ag_2_CO_3_ nanoparticles on Bi_2_WO_6_. Figure 5b is the HR-TEM image of Ag_2_CO_3_/Bi_2_WO_6_. the lattice spacing of 0.252 nm corresponds to the (− 2 0 1) crystal plane of Ag_2_CO_3_, and the lattice spacing of 0.207 nm corresponds to the (1 1 2) crystal plane of Bi_2_WO_6_. This is further evidence that the AB heterojunction was successfully constructed.

**Figure 6:**
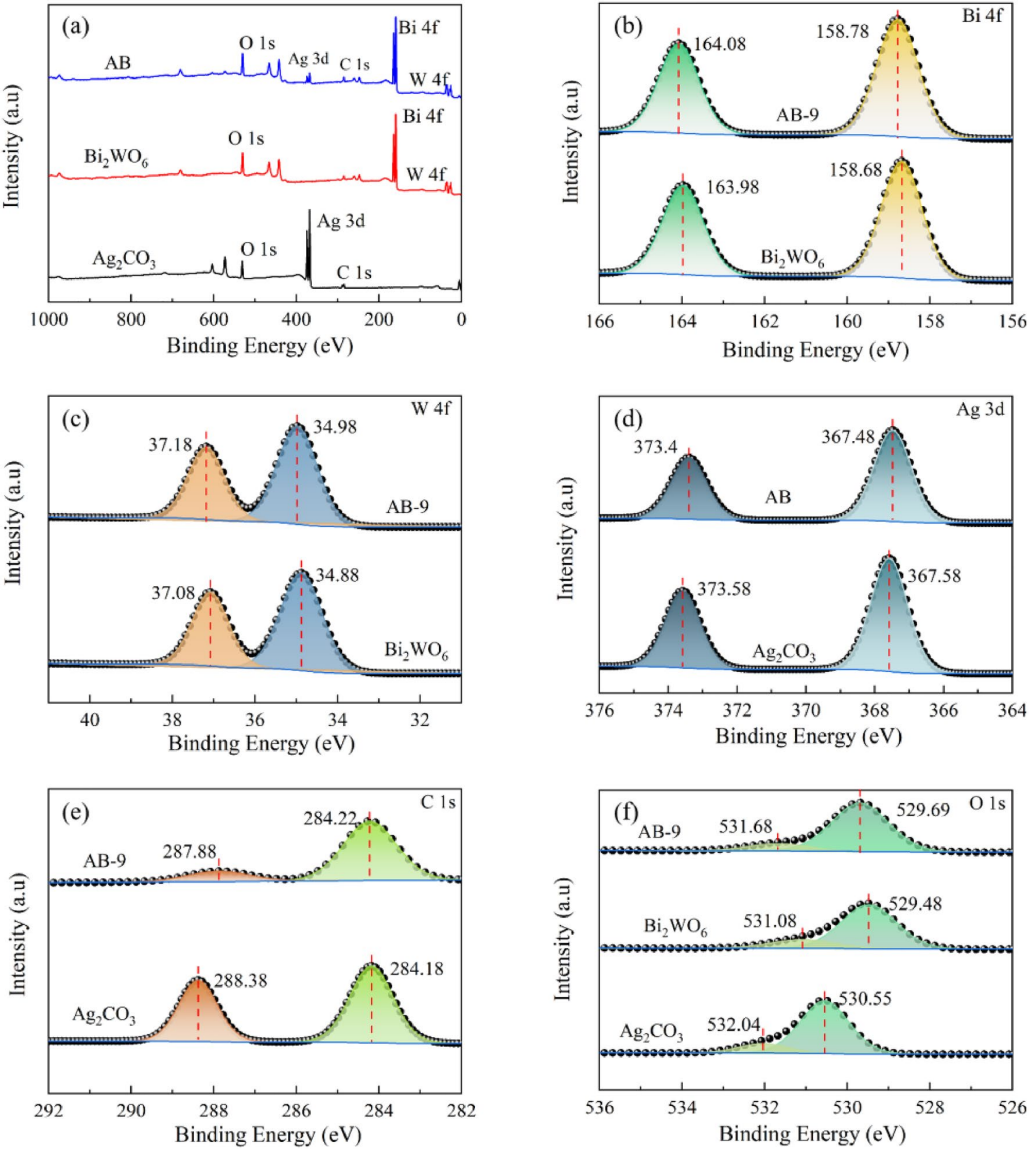
(**a**) Full elemental spectra of photocatalysts, (**b**–**f**) Fine spectra of Bi, W, Ag, C and O elements.

The chemical composition and elemental valence states of the AB-9 surface were analyzed by the XPS technique. Figure 6a displays the presence of Ag, C, O, Bi, and W elements in the nanocomposite AB-9, consistent with the EDS analysis. The fine spectra of Ag, C, O, Bi, and W elements are shown in Fig. 6b–f, respectively. In Bi_2_WO_6_, the characteristic peaks of Bi 4*f*. are observed as two strong peaks at 163.98 eV (Bi 4*f*_7/2_) and 158.68 eV (Bi 4*f*_5/2_), indicating the presence of Bi^3+^ ions^35^. The two independent peaks of W 4*f*. are 37.08 eV (W 4*f*_5/2_) and 34.88 eV (W 4*f*_7/2_), suggesting the existence of W^6+^ ions^36^. Comparatively, in the AB-9 nanocomposite, the binding energies of both Bi 4*f*. and W 4*f*. elements are higher than in Bi_2_WO_6_, suggesting that both Bi and W elements in Bi_2_WO_6_ lost electrons during the composite process with Ag_2_CO_3_.

In Ag_2_CO_3_, the Ag 3*d* spectrum exhibits two independent peaks of 373.58 eV (Ag 3*d*_3/2_, Ag^+^) and 367.58 eV (Ag 3*d*_5/2_, Ag^+^)^22,37^. The C 1*s* spectrum displays two distinct characteristic peaks of 288.38 and 284.18 eV, with the former mainly attributed to the C=O bonding in Ag_2_CO_3_, and the latter to the amorphous carbon^38^. By contrast, the binding energies of both Ag 3*d* and C 1*s* in the AB-9 are reduced. The O 1*s* spectrum of Bi_2_WO_6_ exhibits two peaks of 529.48 and 531.08 eV, corresponding to oxygen in the Bi–O and W–O bonds, respectively^39^. In the O 1*s* spectrum of Ag_2_CO_3_, the characteristic peak at 530.55 eV is attributed to the lattice oxygen, while the characteristic peak at 532.04 eV belongs to the adsorbed oxygen^40^. The increase in the binding energy of O 1*s* in the AB-9 suggests that the elemental oxygen loses electrons, possibly due to the changes in work function, lattice potential electronegativity difference, and other factors. However, undoubtedly, this phenomenon also confirms the existence of interaction between Bi_2_WO_6_ and Ag_2_CO_3_. In general, the binding energy of Bi_2_WO_6_ is shifted to the high-energy region, while that of Ag_2_CO_3_ is shifted to the low-energy region, indicating that the electron density of Bi_2_WO_6_ is lower and that of Ag_2_CO_3_ is higher within the AB-9. This also suggests that the electrons are transferred from Bi_2_WO_6_ to Ag_2_CO_3_ in the AB-9 upon contact between the two monomers. These results unequivocally demonstrate the successful combination of the two materials, and the formation of a heterojunction.

### Photocatalytic performance of AB for LEV

**Figure 7:**
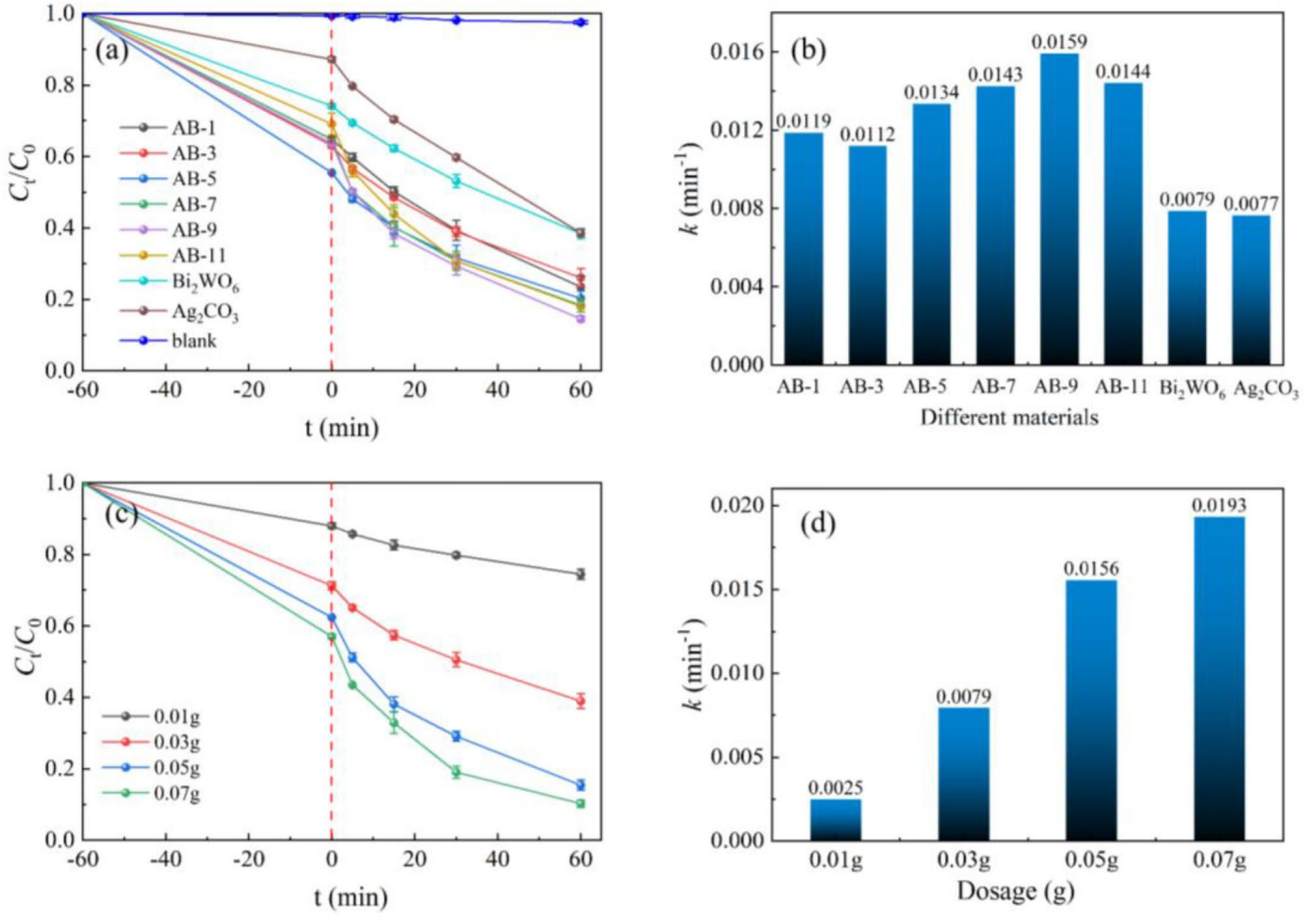
(**a**, **b**) Degradation of LEV with different materials and degradation rate constant *k*, (**c**, **d**) Degradation of LEV with different AB-9 dosage and degradation rate constant *k*.

The photocatalytic degradation performance of AB nanocomposites was assessed by examining the degradation effect on LEV under visible light irradiation. Figure 7a depicts the photocatalytic degradation rates of AB with varying mass fractions of Ag_2_CO_3_ for LEV. The experimental results showed that the LEV solution was stable without photocatalyst and did not decompose under visible light irradiation. Upon the addition of the photocatalyst to the LEV solution and subsequent stirring under dark conditions for 1 h, the reaction system reached the adsorption–desorption equilibrium. The specific details of dark adsorption are presented in Figure S1. At this time, the removal of LEV from the solution by Bi_2_WO_6_ and Ag_2_CO_3_ was determined to be 25.84 and 12.98%, respectively. This indicates that these two monomers exhibit superior adsorption capabilities for LEV, thereby providing abundant reaction sites in the photocatalytic degradation process. Following 60 min of xenon lamp irradiation, the degradation efficiencies of LEV solutions using Bi_2_WO_6_ and Ag_2_CO_3_ as photocatalysts were determined to be 61.56 and 61.4%, respectively. Notably, the degradation rate of LEV was significantly enhanced when nanocomposites containing different mass fractions of Ag_2_CO_3_ were used as photocatalysts. The degradation rates of AB-1, AB-3, AB-5, AB-7, AB-9, and AB-11 reached 76.49%, 73.89%, 79.67%, 81.61%, 85.4%, and 81.92%, respectively, with AB-9 exhibiting the highest photocatalytic capability during the process, surpassing that of the individual monomers. In the case of single photocatalysts, the recombination of e^−^ and h^+^ was prone to occur under light conditions, resulting in lower photocatalytic activities of Bi_2_WO_6_ and Ag_2_CO_3_. However, when the two photocatalysts were combined to form a heterojunction, they exhibited an enhanced capacity for visible light and reduced the recombination of photogenerated carriers. The degradation rate of LEV by AB is significantly increased, confirming the beneficial effect of the heterojunction formed between Ag_2_CO_3_ and Bi_2_WO_6_ in improving the photocatalytic degradation process.

The degradation curve of LEV was fitted with the quasi-first-order kinetic equation -Ln(*C*_t_/*C*_0_) = *kt*, as shown in Fig. 7b. Here, *C*_0_ and *C*_t_ represent the initial and instantaneous concentrations of LEV (mg/L), respectively, and *k* denotes the reaction kinetic constant that signifies the rate at which the degradation proceeds. The degradation rate constants for Bi_2_WO_6_, Ag_2_CO_3_, AB-1, AB-3, AB-5, AB-7, AB-9, and AB-11 were 0.0079, 0.0077, 0.0119, 0.0112, 0.0134, 0.0143, 0.0159, 0.0144 min^−1^, respectively. The variation in degradation rate aligns with the catalytic activity of the photocatalysts. AB-9 exhibited the highest degradation rate, which was approximately twofold that of Bi_2_WO_6_ and Ag_2_CO_3_ monomers.

AB-9, which has the best photocatalytic degradation effect, was employed as a photocatalyst to investigate the impact of AB dosage on LEV degradation. Figure 7c elucidates the influence of AB-9 dosage on the degradation rate of LEV. The degradation rate of LEV reached 84.55% when the dosage of AB-9 was increased from 0.01 to 0.05 g. However, with further increases in dosage, the degradation rate only exhibited a marginal improvement of 5.25%, reaching 89.8%. Similarly, the corresponding photocatalytic degradation rate constant *k*, as shown in Fig. 7d, exhibits a six-fold change with the addition of photocatalyst from 0.01 to 0.05 g, and a smaller change from 0.05 to 0.07 g. Although, increasing the photocatalyst dosage provides more active sites for the photocatalytic degradation reaction, excessive amounts of photocatalyst may hinder the transmission of light paths in the solution. This can impede its absorption ability for visible light and hinder the incident light irradiation from reaching the solution’s interior. Consequently, the number of photogenerated carriers decreases, thereby affecting the photocatalytic degradation performance of AB-9.

**Figure 8:**
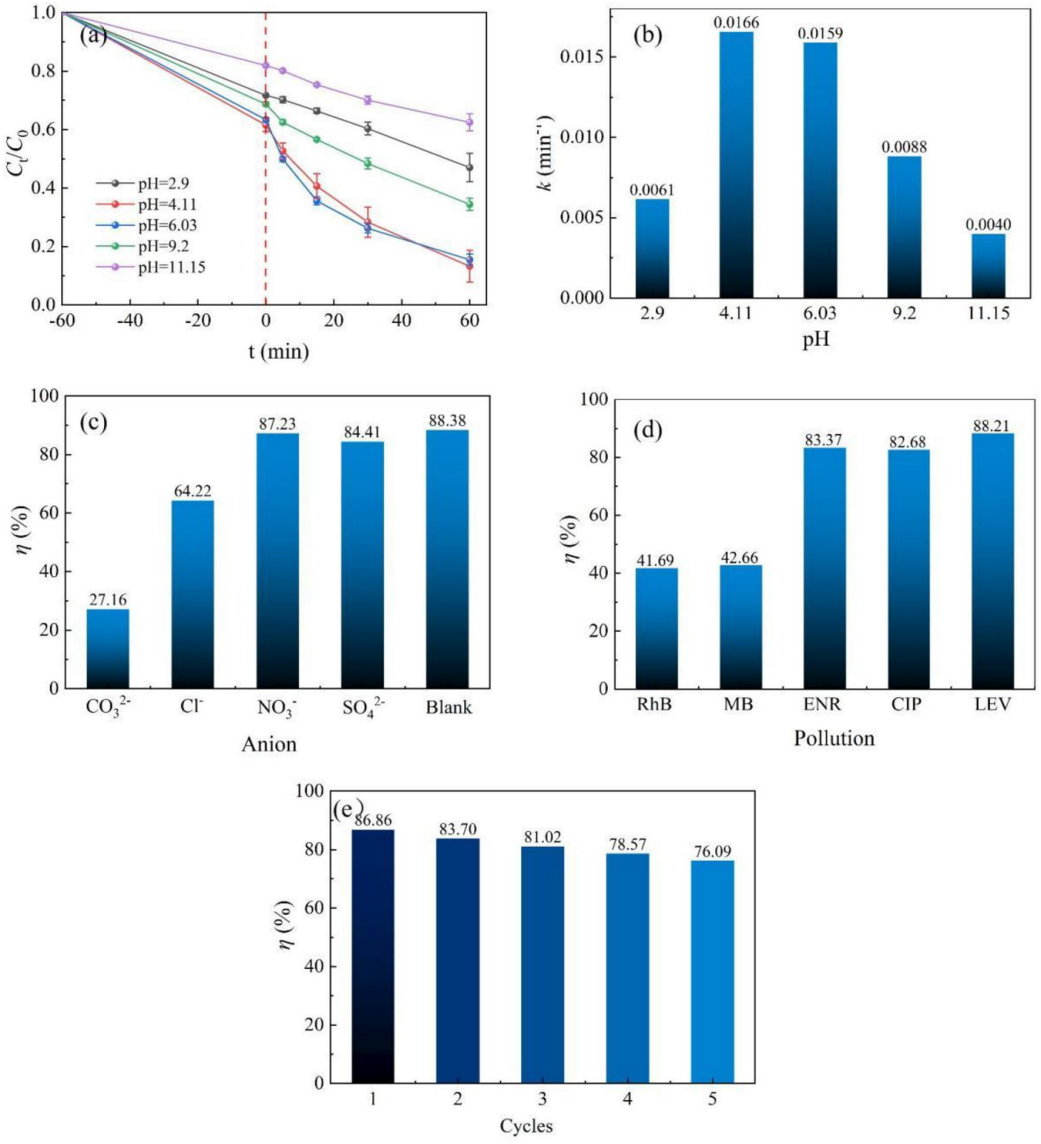
(**a**, **b**) Degradation of LEV by AB-9 and degradation rate constants *k* at different pH, (**c**) Degradation rate of LEV by AB-9 with different anions, (**d**) Degradation rate of different pollutants by AB-9 (**e**) reusability of AB-9.

The pH of the solution is the main factor influencing the photocatalytic degradation of organic pollutants. Figure 8a and b shows the photocatalytic effect of AB-9 on LEV and the degradation rate constant *k* at varying pH. At pH 4.11 and 6.03, the degradation rate of LEV reached 86.72 and 84.48%, respectively, accompanied by the high degradation rate constants *k*. These results indicate that LEV can be degraded rapidly under environmental pH, showing excellent photocatalytic degradation ability. However, at pH 2.9, the degradation rate of LEV declined to 53%. The decline may be attributed to the fact that e^-^ cannot stabilize under the highly acidic environment, and cannot further react to generate ·O_2_^−^. Moreover, the degradation rate of LEV was 65.56 and 37.51% at pH 9.2 and 11.15, respectively. Under alkaline conditions, h^+^ reacts with hydroxyl to form ·OH, which exhibits poor stability and participates in photocatalytic degradation in small amounts. As the alkalinity of the solution increases, the degradation of LEV further decreases, indicating that h^+^ may play a major role in the degradation process.

Wastewater usually contains a diverse array of inorganic anions, and the environment in which CO_3_^2−^, Cl^−^, NO_3_^−^, and SO_4_^2−^ are present was simulated by adding Na_2_CO_3_, NaCl, NaNO_3_, and Na_2_SO_4_ to the solution. Figure 8c illustrates the effect of different anions on the photocatalyst degradation of LEV. Experimental results showed that the addition of SO_4_^2−^ and NO_3_^−^ had minimal influence on the photocatalytic performance of the catalyst. However, the addition of Cl^−^ reduced the photocatalytic ability to 64.22%. It is commonly believed that Cl^−^ readily adsorbs on the catalyst surface, impeding the adsorption of reactant molecules and thereby inhibiting the reaction^41^. Following the addition of CO_3_^2−^, the photocatalyst achieved a mere 27.16% degradation of LEV. This can be attributed to the reaction of h^+^ with CO_3_^2−^ generated by visible light excitation catalyst, resulting in h^+^ quenching and rendering it ineffective in the photocatalytic degradation reaction.

To further investigate the practical applicability of photocatalyst AB-9, several common pollutants were selected for degradation testing, and the results are shown in Fig. 8d. Under the same conditions employed for the photocatalytic degradation of LEV, AB-9 exerted a better degradation effect towards enrofloxacin (ENR) and ciprofloxacin (CIP), achieving degradation levels of 83.37 and 82.68%, respectively. By contrast, AB-9 exhibited less effectiveness in degrading rhodamine B (RhB) and methylene blue (MB). This disparity can mainly be attributed to the fact that ENR and CIP, being quinolone antibiotics with similar properties and stability as LEV, are more amenable to degradation with AB-9. On the other hand, MB and RhB, as common azo dyes, contain many conjugated structures that required higher energies of reactive radicals for degradation. Therefore, when compared to quinolone antibiotics, the photocatalyst AB-9 displays limited capability in degrading azo dyes.

The stability of the photocatalyst AB-9 was evaluated through the reusability experiment. After five cycles, the degradation activity of photocatalyst AB-9 on LEV exhibited a slight reduction, yet the degradation efficiency was still maintained at approximately 75%, as shown in Fig. 8e. To further explore the stability of the sample, the AB-9 was characterized by SEM (Figure S2) and XPS (Figure S3) before and after the reaction. The morphology and chemical composition of the sample before and after photocatalysis have negligible changes. It is further confirmed that the photocatalyst AB-9 is a relatively stable photocatalytic degradation material. In Table 1, this work is compared with the photocatalyst degradation of LEV reported in other literature. Compared with other degradation work, the photocatalyst prepared in this paper has a better degradation effect on LEV.

**Table 1:** Comparison of some photocatalytic systems degrading LEV.

Photocatalytic system	Antibiotic conc. (10 mg/L)	Catalyst dosage (g/L)	Irradiation time (min)	Photocatalytic efficiency (%)	Refs.
Ag_2_O/P-g-C_3_N_4_/vis	10	1	120	83	^42^
Bi_2_WO_6_ nanocuboids/vis	10	0.75	150	80	^43^
Ag/AgBr/BiOBr/vis	10	1	90	74	^44^
Ag_3_PO_4_/MFe_2_O_4_(M=Zn, Ni, Co)/vis	10	1	60	75.5	^45^
CdO nanoplates/sunlight	5	1	240	80	^46^
Bi–Bi_2_WO_6_/BiOI/vis	20	0.8	60	83.3	^47^
Ag_2_CO_3_/Bi_2_WO_6_/vis	10	1	60	85.4	This work

### Optical properties of nanocomposite AB

**Figure 9:**
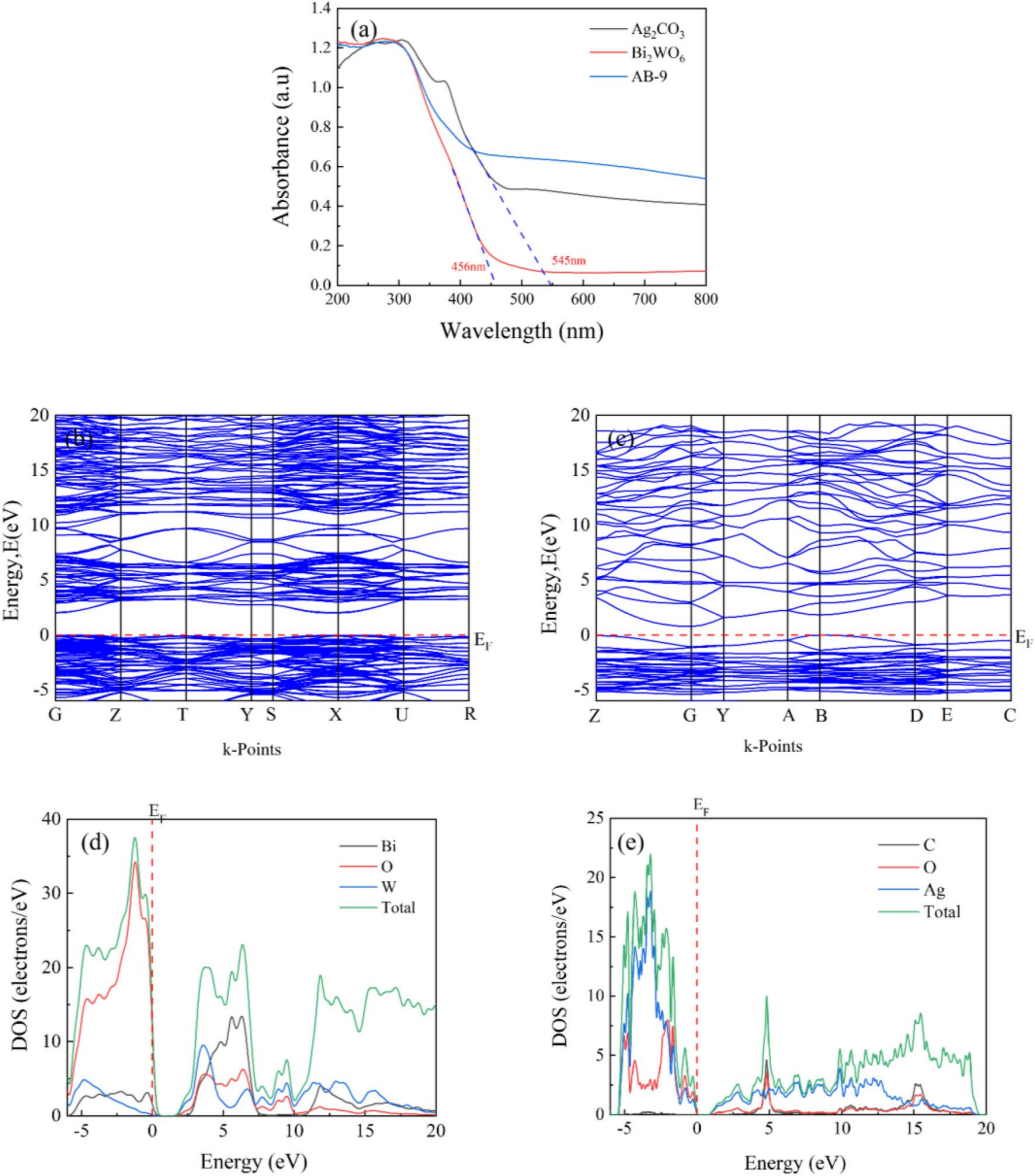
(**a**) UV–vis-DRS of materials, (**b**, **c**) energy band mappings of Bi_2_WO_6_ and Ag_2_CO_3_, (**d**, **e**) DOS of Bi_2_WO_6_ and Ag_2_CO_3_.

To investigate the visible light absorption properties of Bi_2_WO_6_, Ag_2_CO_3_, and nanocomposite AB-9, the UV–vis diffuse reflectance spectroscopy (UV–vis-DRS) was employed for analysis. As shown in Fig. 9a, the absorption threshold of Bi_2_WO_6_ is approximately 456 nm, which indicates limited absorption of visible light, and restricts its photocatalytic performance. On the other hand, Ag_2_CO_3_ exhibits an absorption edge at around 545 nm, indicating a certain degree of visible light absorption capability. Upon combining the two photocatalytic components to form the AB-9 nanocomposite, the visible light absorption ability of the photocatalyst AB-9 is notably improved. This enhancement can be attributed to the synergistic interaction between the two photocatalysts.

Based on the crystal structures of Ag_2_CO_3_ and Bi_2_WO_6_ obtained by XRD analysis, and combined with DFT calculations, the energy bands of Ag_2_CO_3_ and Bi_2_WO_6_ were determined to be 0.779 and 2.019 eV, respectively, as shown in Fig. 9c and b. The highest occupied molecular orbital of Ag_2_CO_3_ is located at the high-symmetry point G, while the lowest unoccupied molecular orbital is situated at the high-symmetry point B, indicating that Ag_2_CO_3_ is an indirect bandgap semiconductor. Conversely, both the highest occupied molecular orbital and the lowest unoccupied molecular orbital of Bi_2_WO_6_ are positioned at the high symmetry point G, making it a direct bandgap semiconductor. It is worth noting that the bandgap obtained through GGA-PBE function tends to be smaller than the actual bandgap^48^. Analyzing the density of states of Bi_2_WO_6_ (Fig. 9d), it becomes evidently the CB is mainly contributed by O and Bi atoms, while the valence band (VB) originates from O and W atoms. On the other hand, examining the density of states of Ag_2_CO_3_ (Fig. 9e), it can be observed that the CB is mainly influenced by O and Ag atoms, while the VB is derived from Ag atoms.

**Figure 10:**
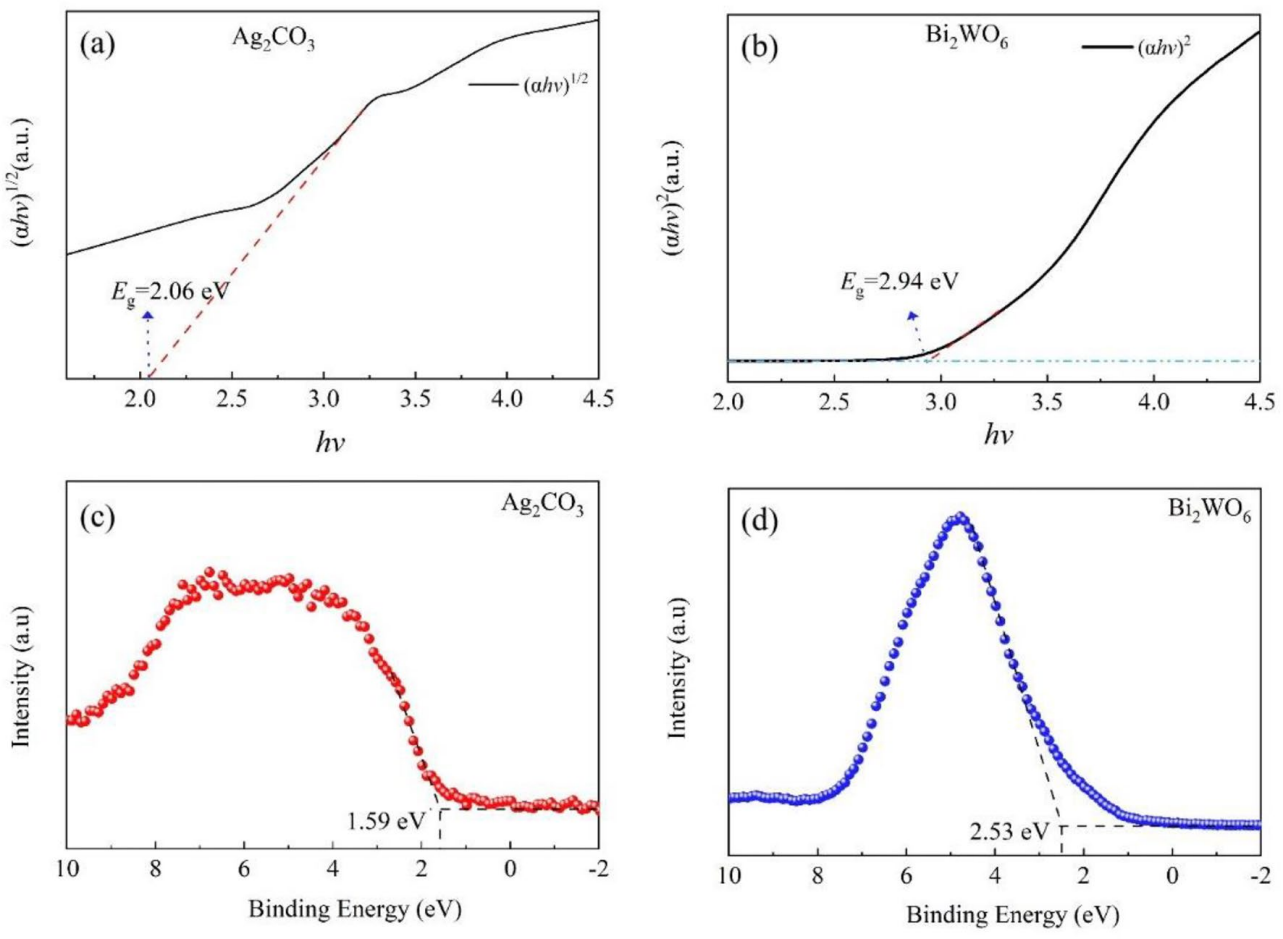
(**a**, **b**) Tauc plots of Ag_2_CO_3_ and Bi_2_WO_6_, (**c**, **d**) VB-XPS of Ag_2_CO_3_ and Bi_2_WO_6._

To accurately determine the bandgap of the photocatalyst, the UV–vis-DRS data was processed using that Tauc equation *αhυ* = A (*hυ*–*E*_g_)^*n*/2^. Where *α* is the absorption constant, *h* is Planck’s constant, *υ* is the optical frequency, A is the proportionality constant, *E*_g_ is the bandgap, and *n* is a constant associated with the semiconductor’s carrier leaps. For direct semiconductors, *n* = 1, while for indirect semiconductors, *n* = 4. The Tauc plots for Ag_2_CO_3_ and Bi_2_WO_6_, are shown in Fig. 10a and b. From these plots, it can be concluded that the band gaps of Ag_2_CO_3_ and Bi_2_WO_6_ are 2.06 and 2.94 eV, respectively. The valence band X-ray photoelectron spectra (VB-XPS) are presented in Fig. 10c and d, where the VB values of Ag_2_CO_3_ and Bi_2_WO_6_ are 1.59 and 2.53 eV, respectively. Additionally, the calculation yields the CB values of Ag_2_CO_3_ and Bi_2_WO_6_ as − 0.47 and − 0.41 eV, respectively.

**Figure 11:**
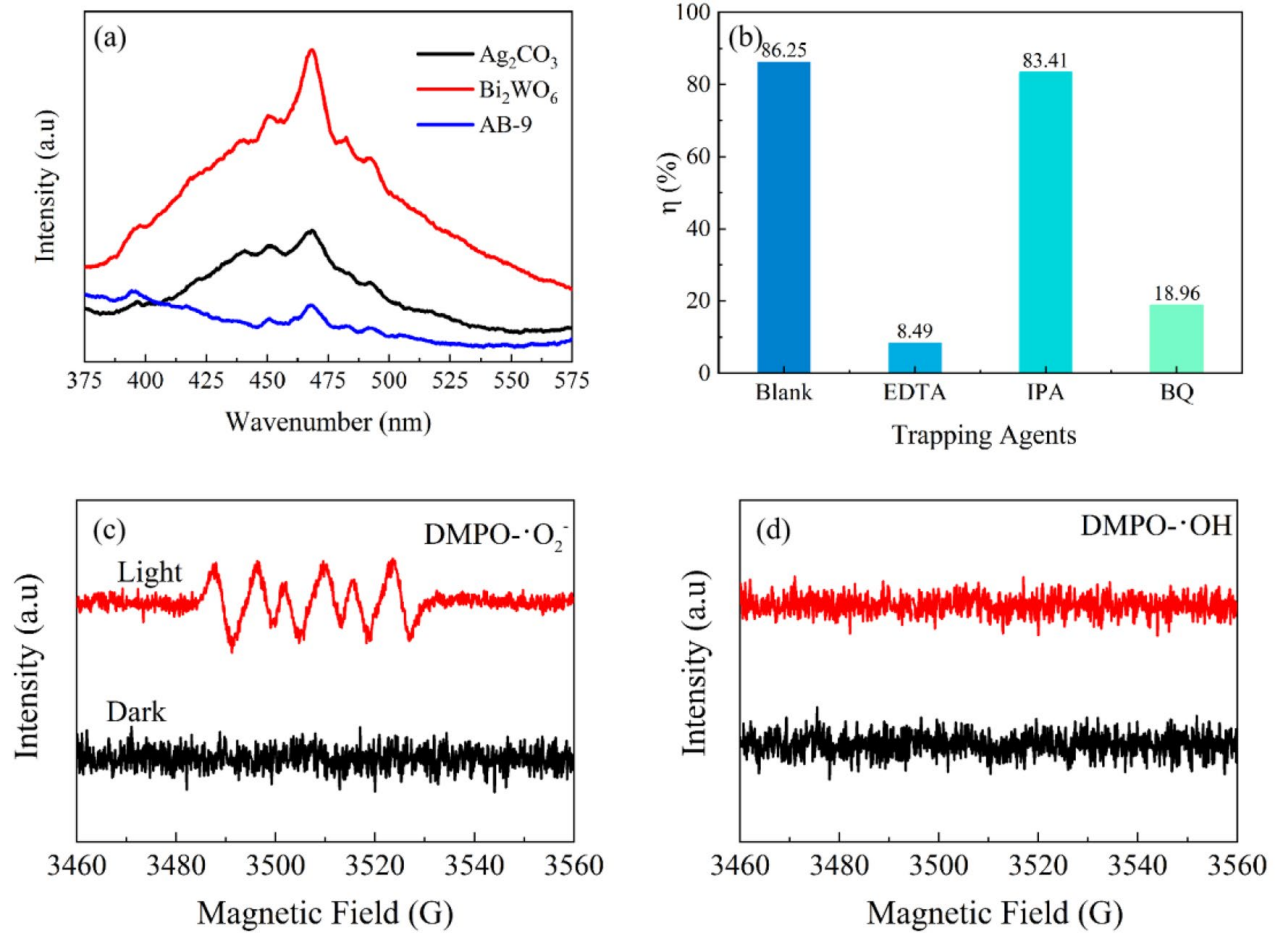
(**a**) Fluorescence spectra of materials, (**b**) Effect of trapping agents on LEV degradation, (**c**) ESR of DMPO-·O_2_^−^, (**d**) ESR of DMPO-·OH.

The carrier separation efficiency was investigated using fluorescence spectroscopy, and the results are shown in Fig. 11a ^49^. The findings show that the emission peaks of both Bi_2_WO_6_ and Ag_2_CO_3_ monomers are higher than those of the nanocomposite AB. Notably, the emission peak intensity of Bi_2_WO_6_ is the highest, indicating that the recombination of e^−^ and h^+^ pairs within Bi_2_WO_6_ occurs more easily. The emission peak intensity of Ag_2_CO_3_ is relatively low, possibly due to its nature as an indirect bandgap semiconductor. Comparatively, the nanocomposite AB-9 exhibits a reduced fluorescence intensity in comparison to Ag_2_CO_3_ and Bi_2_WO_6_ monomers. This suggests that the heterojunction formed between Ag_2_CO_3_ and Bi_2_WO_6_ significantly mitigates the carrier recombination rate. Subsequently, to further investigate the photoelectron transfer rate, EIS plots of Ag_2_CO_3_, Bi_2_WO_6_ and AB-9 were constructed in 0.1 Hz-0.1 M Hz. The equivalent circuit for the system is presented in the inset image of Figure S4. In this model, R_0_, R_2_, W_3_, and C_1_ respectively represent the resistance between the fluorine-doped tin oxide and catalysts, the charge migration resistance across the photoanode/electrolyte interface, the Warburg impedance, and the constant phase element^50^. In principle, a smaller EIS radius and R_2_ represent the faster the charge migration rate. In the EIS plots, AB-9 manifests a smaller arc radius and the lower R_2_ of 3933 Ω compared with Ag_2_CO_3_ (R_2_, 4031 Ω) and Bi_2_WO_3_ (R_2_, 5203 Ω), indicating the lowest charge-migration resistance and the best charge transfer effect.

### Photocatalytic mechanism

To elucidate the degradation mechanism of the AB-9 nanocomposite on LEV under visible light, free radical capture experiments were conducted to identify the key free radicals involved in the degradation process. Scavenging of ·OH, ·O_2_^−^ and h^+^ was performed with 5 mM of IPA, BQ and EDTA, respectively. The results, as seen in Fig. 11b, demonstrate that the degradation rate reached 86.25% when only the photocatalyst was added to the LEV solution. Upon the addition of EDTA, the degradation rate of LEV decreased significantly to 8.49%, indicating a 78% reduction. This confirms the crucial role of h^+^ in the degradation process of LEV. Furthermore, the introduction of BQ resulted in a 67% decrease in the degradation rate of LEV, indicating that ·O_2_^−^ serves as a secondary reactive radical in the degradation process. When IPA was added, the degradation rate of LEV was unchanged, indicating that ·OH had little influence on the degradation process. In summary, the nanocomposite AB mainly employs h^+^ and ·O_2_^−^ as the main contributors to the degradation of LEV.

To further explore the photocatalytic reaction mechanism, the ESR spectroscopy was carried out to determine the active species generated from the photocatalytic system with AB-9 as the photocatalyst, as shown in Fig. 11c and d. It should be noted that no peaks of DMPO- ·O_2_^−^ and DMPO- ·OH are found according to the test data in the dark. However, upon 5 min of visible light irradiation, strong peak signals with an intensity ratio of 1:1:1:1 for DMPO- ·O_2_^−^ were detected, indicating the generation of ·O_2_^−^ radicals^51^. Furthermore, the DMPO-·OH spectrum fails to exhibit its characteristic peaks, so the process does not produce ·OH radicals. This result is consistent with that of the free radical capture experiment.

**Figure 12:**
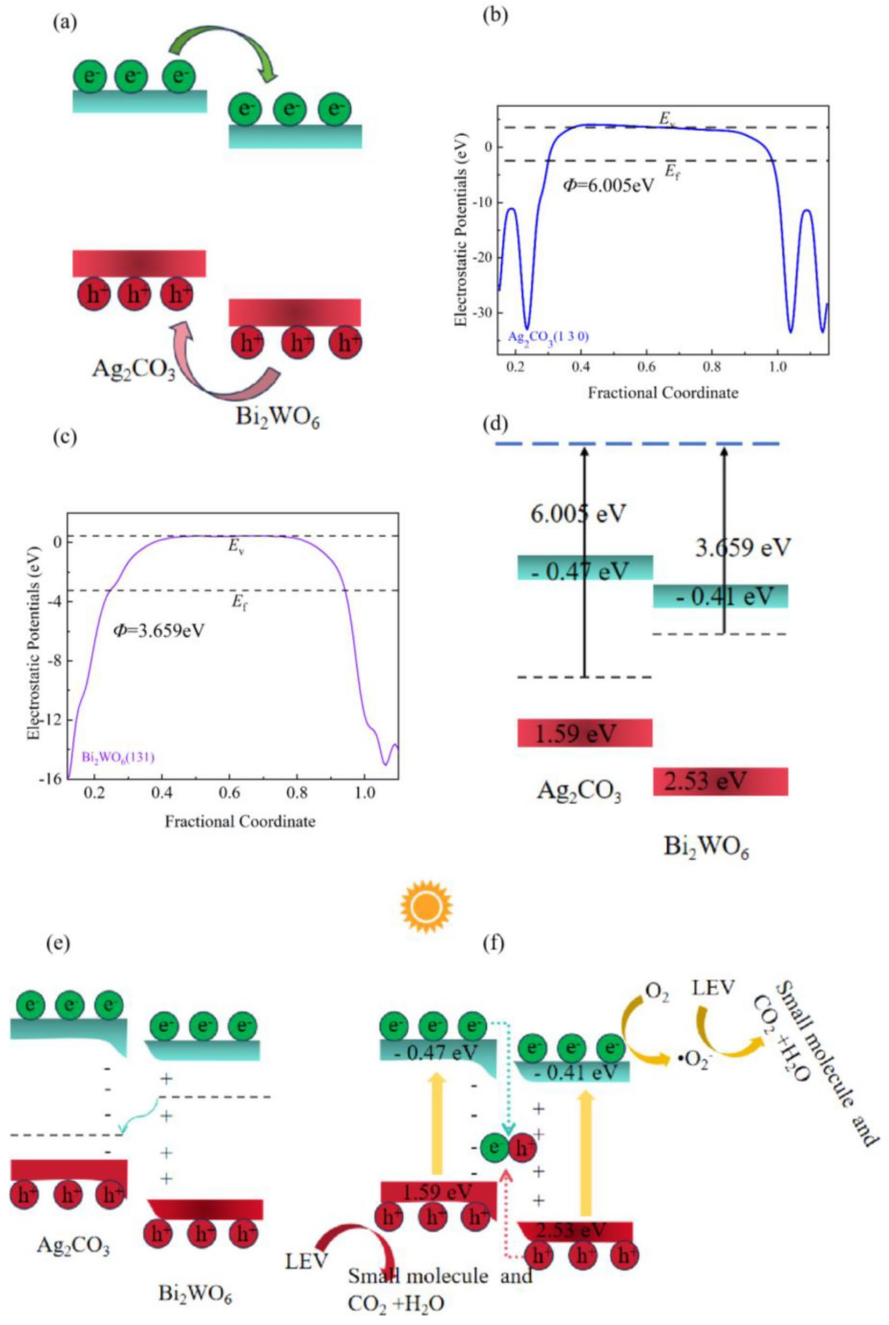
(**a**) Conventional illustration of type II Heterogeneous, (**b**, **c**) Work functions of Ag_2_CO_3_ and Bi_2_WO_6_, (**d**–**f**) Formation conditions of the IEF and the carrier transfer in AB.

Based on the energy band distribution of the AB nanocomposite, it can be assigned to a type-II heterojunction. The conventional interpretation of the photogenerated carrier transfer path for type II heterojunction is shown in Fig. 12a. According to this interpretation, e^−^ in the CB of Ag_2_CO_3_ drifts to the CB of Bi_2_WO_6_, while h^+^ in the VB of Bi_2_WO_6_ drifts to the VB of Ag_2_CO_3_, thus completing the separation of the photogenerated carriers. However, from the kinetic point of view, the repulsive forces between e^−^ (or between h^+^) can hinder the transfer of the aforementioned assumptions.

In recent years, the influence of the IEF on the transfer of photogenerated carriers has been recognized by many researchers. It has been mainly employed to explain the cases involving heterojunctions formed by a semiconductor with both a higher CB and a higher *E*_f_ compared to another semiconductor, and type-II–I is appropriate for this. For type-II–II heterojunctions such as AB, where the photocatalytic performance can be improved compared to the monomer despite the CB and *E*_f_ of Ag_2_CO_3_ not being simultaneously higher than those of Bi_2_WO_6_, we attempted to provide a complementary analysis of the charge transfer mechanism of type-II–II heterojunctions based on the effect of the IEF on carrier migration.

The work function (*Φ*) of a material represents the work done for an electron to move from the *E*_f_ to the vacuum energy level (*E*_V_). DFT calculations reveal that *Φ* is 6.005 and 3.659 eV for the Ag_2_CO_3_ (1 3 0) and Bi_2_WO_6_ (1 3 1) facets, respectively, as shown in Fig. 12b and c. The *Φ* of the Ag_2_CO_3_ (1 3 0) facet is higher than the Bi_2_WO_6_ (1 3 1) facet, indicating that the *E*_f_ of Ag_2_CO_3_ is lower than that of Bi_2_WO_6_ (Fig. 12d). When Ag_2_CO_3_ and Bi_2_WO_6_ form a heterojunction, e^−^ spontaneously transfers from Bi_2_WO_6_ to Ag_2_CO_3_ due to the different *E*_f_ (Fig. 12e). As the *E*_f_ of the two materials reach equilibrium, an electron depletion layer is formed on the Bi_2_WO_6_ side, and an electron accumulation layer is formed on the Ag_2_CO_3_ side, resulting in the formation of an IEF pointing from Bi_2_WO_6_ to Ag_2_CO_3_. The IEF distribution leads to the recombination of e^−^ on the Ag_2_CO_3_ CB and h^+^ on the Bi_2_WO_6_ VB, meanwhile retaining the e^−^ on the Bi_2_WO_6_ CB and h^+^ on the Ag_2_CO_3_ VB, which have a relatively low redox capacity (Fig. 12(f)). The CB potential of Bi_2_WO_6_ has a more negative value than that of O_2_/·O_2_^−^ (− 0.33 eV), allowing the e^−^ on the CB of Bi_2_WO_6_ to easily react with O_2_ (ad.) to form ·O_2_^−^. On the other hand, the VB potential of Ag_2_CO_3_ is not more positive than ·OH/OH^−^ (2.23 eV), preventing the VB of Ag_2_CO_3_ from generating ·OH and participating in the photocatalytic reaction. Eventually, LEV was effectively degraded by ·O_2_^−^ and h^+^.

### Possible degradation pathways

**Figure 13:**
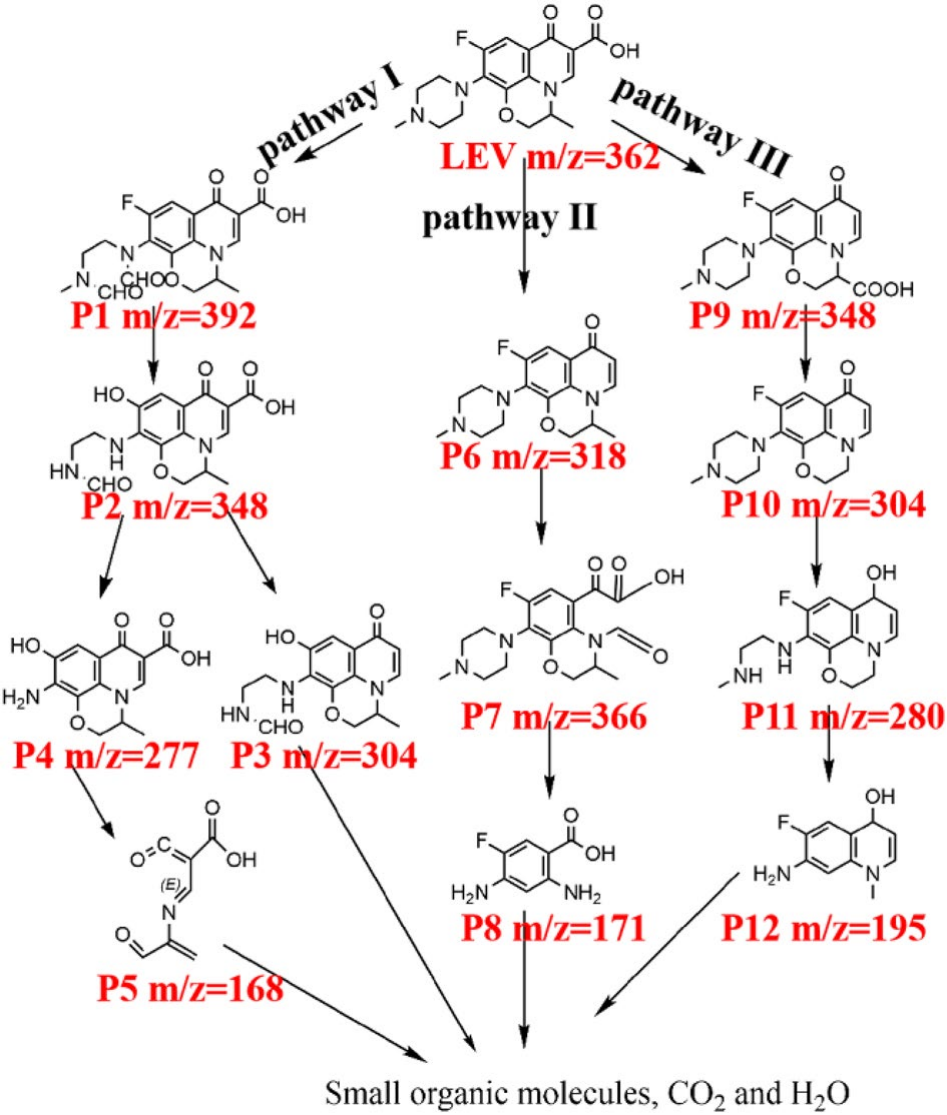
Feasible degradation pathways of LEV onto AB-9.

To elucidate the photocatalytic degradation process of target pollutants, the intermediate products were further determined by high-performance liquid chromatography mass spectrometer (LC–MS). By analyzing the obtained results and considering previous studies, three possible photodegradation routes for LEV are depicted in Fig. 13 (detailed intermediates molecular formula seen in Table S1 and Figure S7). In pathway I, P1 is formed by piperazine ring cleavage of LEV (m/z = 362), and P2 is formed after dialdehyde group and defluorination. P2 may have been completely removed by decarboxylation or piperazine rings to produce P3 or P4. After quinolone ring cleavage and a series of reactions, P4 generates P5. In Pathway II, P6 is formed by decarboxylation of LEV. Subsequently, the quinolone ring in P6 is cleaved to form P7. Piperazine ring cleavage and demethylation form P8. In Pathway III, LEV is decarboxylated first, and then methyl groups are oxidized to form carboxyl groups, giving P9. After further decarboxylation, P10 is formed. After epoxidation of piperazine and cracking of the morpholine ring, P11 and P12 were formed. Finally, all the intermediates are mineralized into small organic molecules, carbon dioxide, and water after a multi-step oxidation reaction, and the photocatalytic degradation of LEV by AB-9 is achieved. Furthermore, the removal of total organic carbon (TOC) was studied (Figure S6). After the photocatalytic reaction for 60 min, the mineralization rate of the solution can reach 42.5%, which further indicates that the LEV can be finally completely mineralized into carbon dioxide and water.

## Conclusion

In this paper, type-II heterojunction is classified into type-II–I and type-II–II considering the relative positions of CB and *E*_f_ in semiconductors. The transfer mechanism of photogenerated carriers in type-II–II heterojunction was investigated through the photocatalytic degradation of LEV by Ag_2_CO_3_/Bi_2_WO_6_ heterojunction.

The results show that the degradation rate of AB-9 to LEV reached 85.4% under the condition of visible light irradiation in 60 min. The degradation mechanism is illuminated by DFT calculation, which confirms the e^−^ in Bi_2_WO_6_ transfer to Ag_2_CO_3_ at the heterojunction interface because the *E*_f_ (− 3.659 eV) of Bi_2_WO_6_ is higher than that of Ag_2_CO_3_ (− 6.005 eV). This calculation result is also demonstrated by the variation of the binding energy of elements in XPS. When the *E*_f_ is equal at the heterojunction interface, the electron depletion layer and accumulation layer are formed at the interface of Bi_2_WO_6_ and Ag_2_CO_3_, respectively, and thus an IEF is built from Bi_2_WO_6_ to Ag_2_CO_3_. Under the action of the IEF, e^−^ in the CB of Ag_2_CO_3_ is recombined with h^+^ in the VB of Bi_2_WO_6_, meanwhile, h^+^ in the VB of Ag_2_CO_3_ and e^−^ in the CB of Bi_2_WO_6_ is retained. The results of fluorescence spectra and EIS on the type-II–II heterojunction Ag_2_CO_3_/Bi_2_WO_6_ indicate that the separation of photogenerated carriers is significantly improved, which is circumstantial evidence of the formation of the IEF. The degradation ability of Ag_2_CO_3_/Bi_2_WO_6_ heterojunction to azo dyes with strong stability is slightly insufficient, which may be caused by the retention of free radicals with the weaker redox ability during the photogenerated carriers migration process in type II–II heterojunctions, and evidence the formation mechanism of the IEF. In general, we propose the classification of type-II heterojunctions and analyze the carrier transfer mechanism of type-II–II heterojunctions, which is an important supplement to the theory of type-II heterojunctions.

### Supplementary Information

Supplementary Information.

## Data Availability

All data that supports the findings of the study are available from the corresponding author with reasonable request.
