# Supplementary material for: A novel type-II–II heterojunction for photocatalytic degradation of LEV based on the built-in electric field: carrier transfer mechanism and DFT calculation
Source: Sci Rep. 2024 May 9;14:10643. doi: 10.1038/s41598-024-60250-z (PMC11082242; doi:10.1038/s41598-024-60250-z)
Supplement: Supplementary file 1 — Supplementary Information. [file 41598_2024_60250_MOESM1_ESM.docx]

**Supporting Information**

**A novel Type-II-II heterojunction for photocatalytic degradation of LEV based on the built-in electric field: carrier transfer mechanism and DFT calculation**

Jiaquan Li ^1^ Peng Tu ^1^ Qian Yang^1^ Yanjun Cui^1^ Chenyang Gao^1^

Hui Zhou^2^ Jun Lu^1^ Hongxia Bian^1^ *

1. College of Science, Gansu Agricultural University, Lanzhou, 730070, PR China

2. School of materials science and Engineering, Lanzhou University of Technology, Lanzhou, 730050, PR China

**
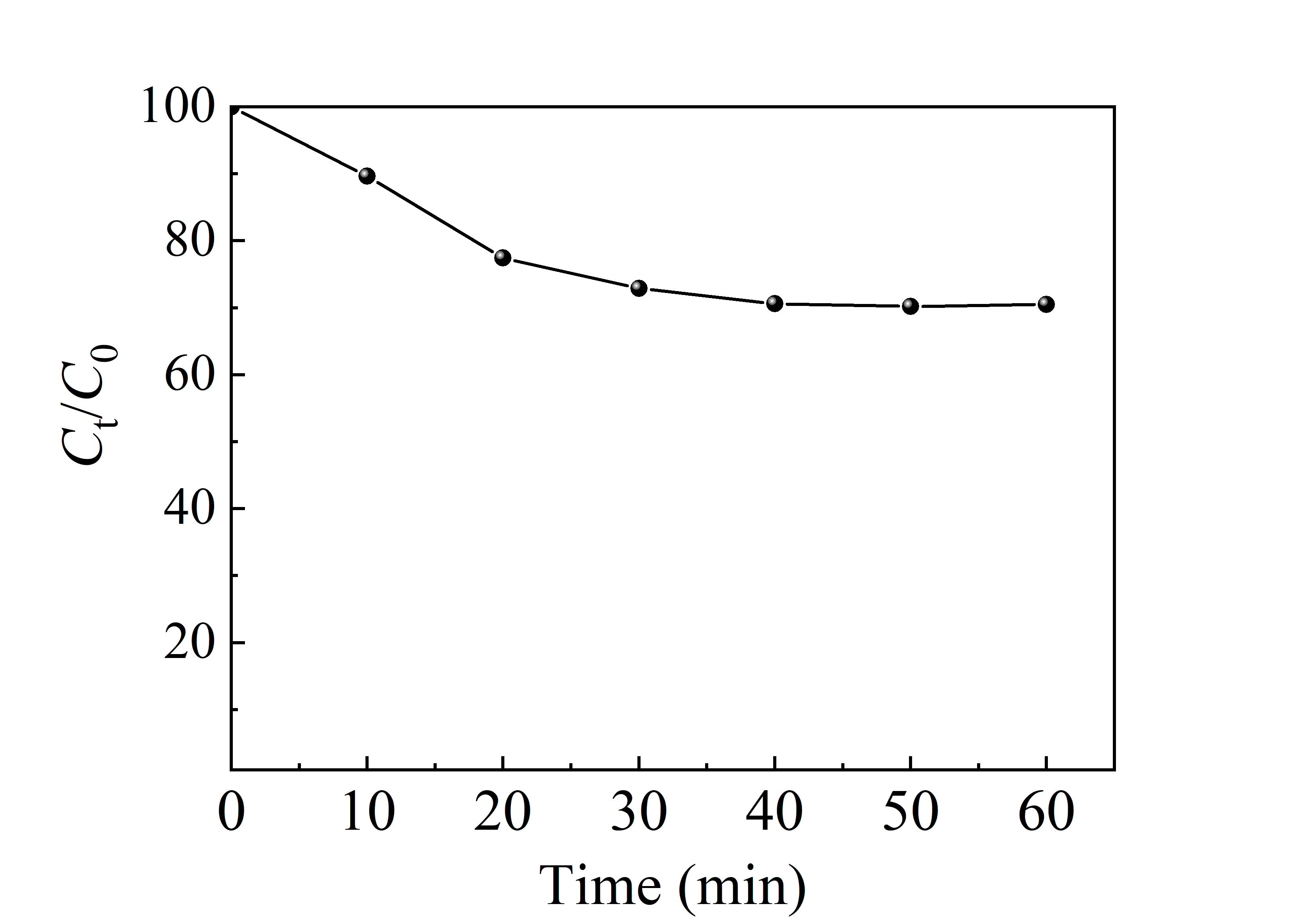
**

Figure S1 Adsorption for LEV of photocatalysts AB-9 under dark conditions.

As displayed in Fig.S1, the adsorption efficiencies of the photocatalyst reached 29% after 30 min of adsorption. The adsorption efficiencies of 60min is 1%, which is only 1% higher than that of 30min, indicating that the whole system has reached the equilibrium of adsorption and desorption at this time. In order to minimize the error, the dark adsorption time is set to 60min.

**
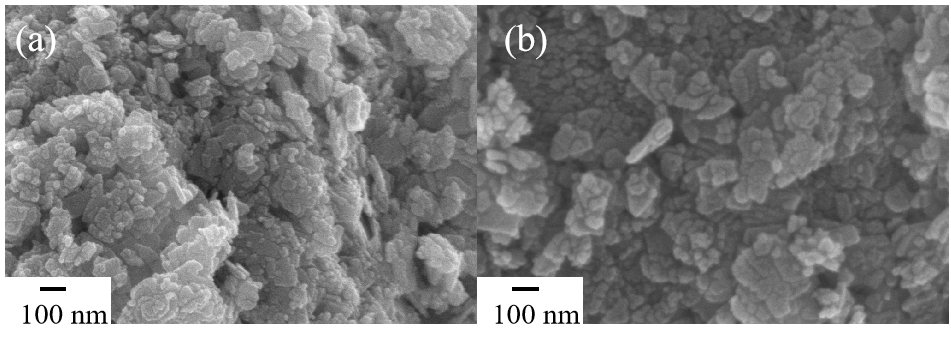
**

Figure S2 The SEM images of AB-9 after photocatalytic reaction.


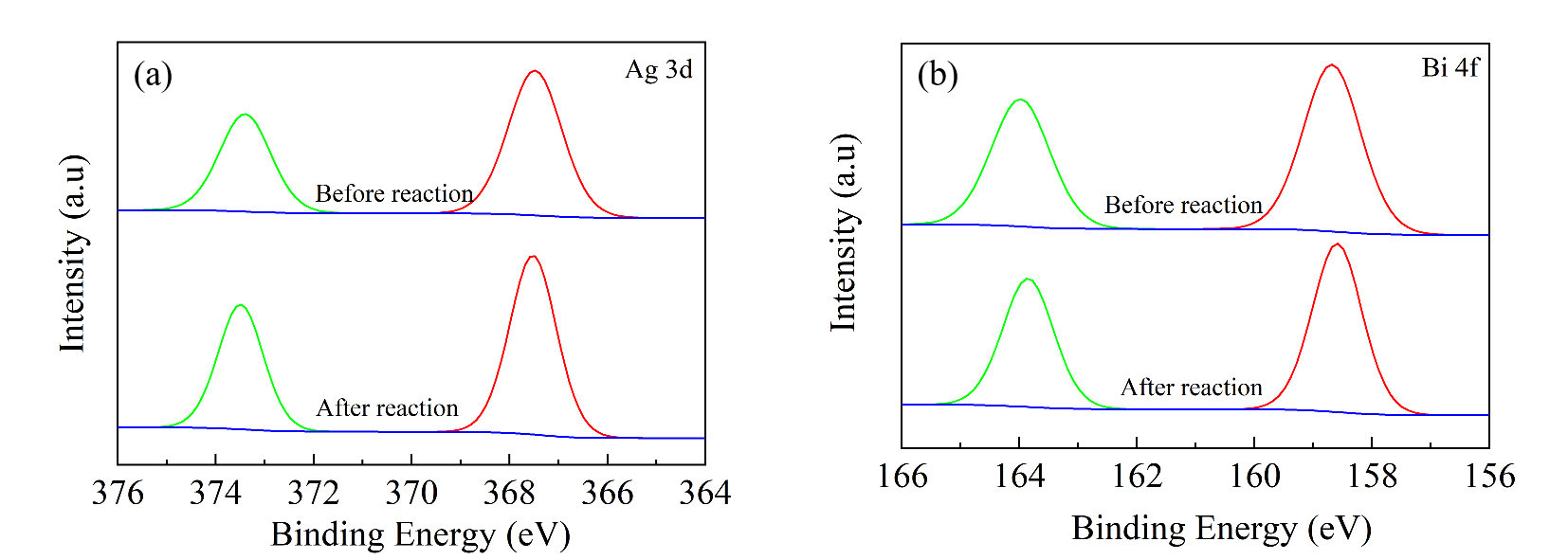


Figure S3 XPS Fine spectra of Bi and Ag elements before and after photocatalytic reaction.


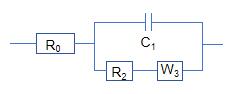
**
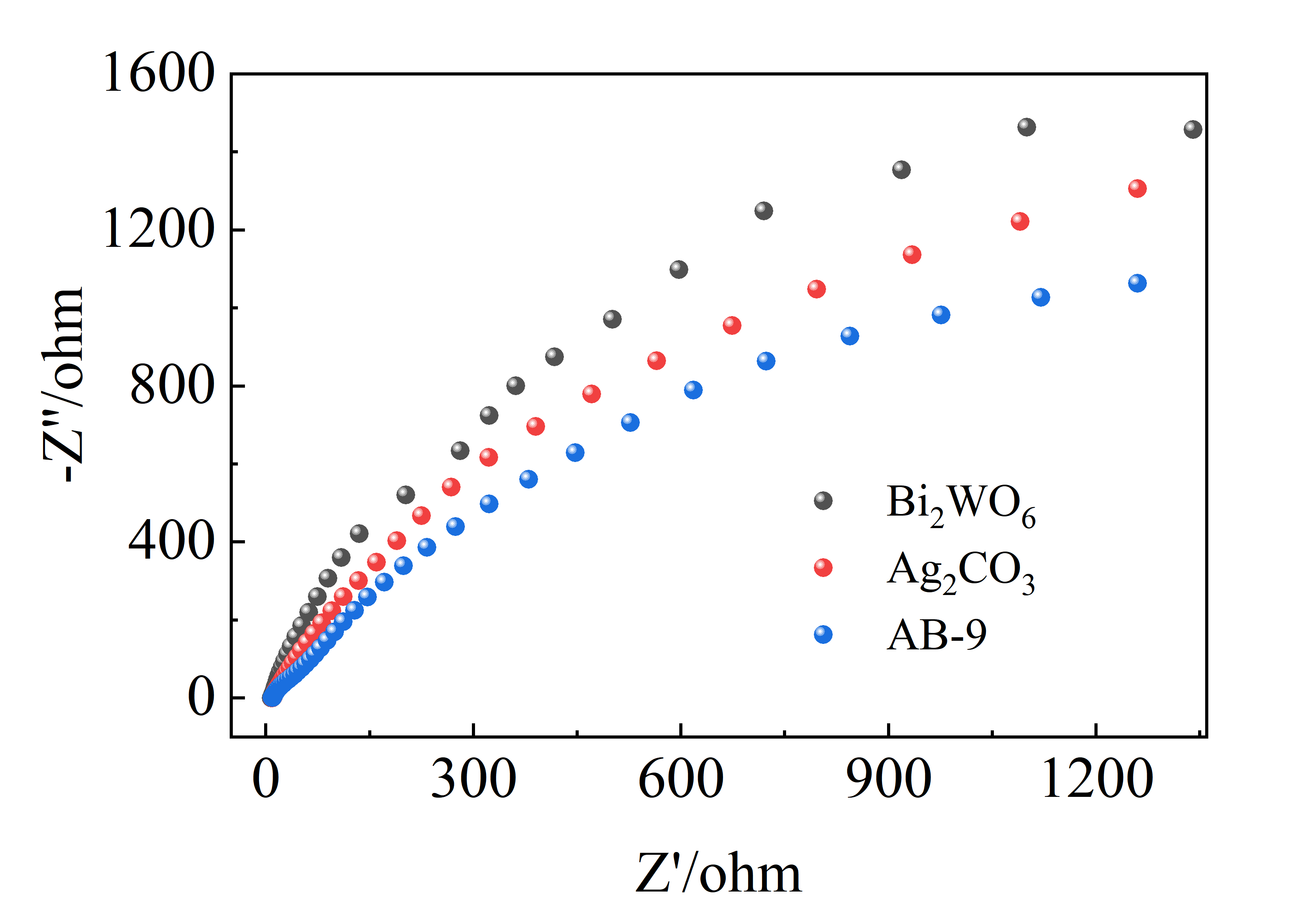
**

Figure S4 EIS of Bi_2_WO_6_, Ag_2_CO_3_, AB-9


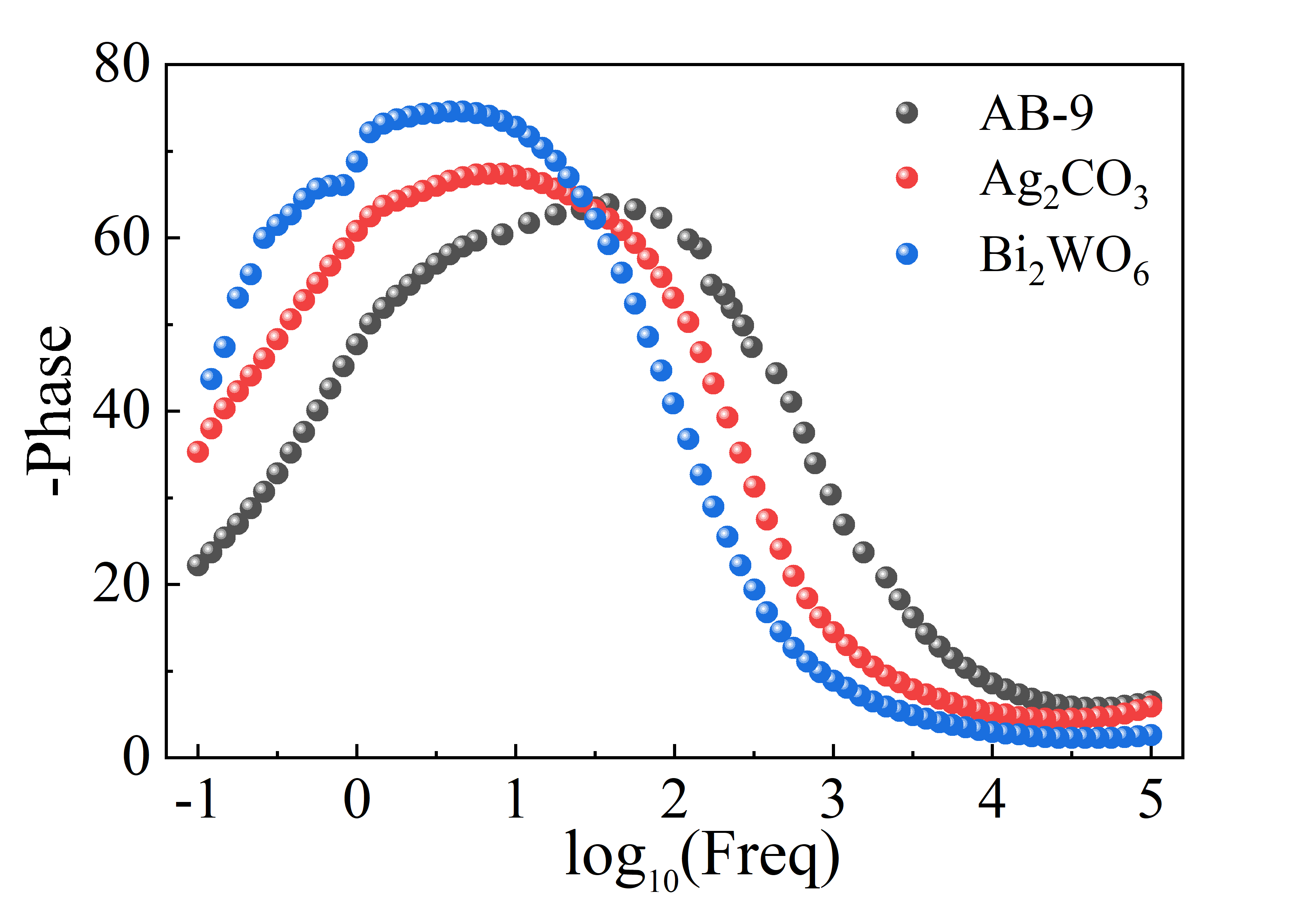


Figure S5. Bode of Bi_2_WO_6_, Ag_2_CO_3_, AB-9

**
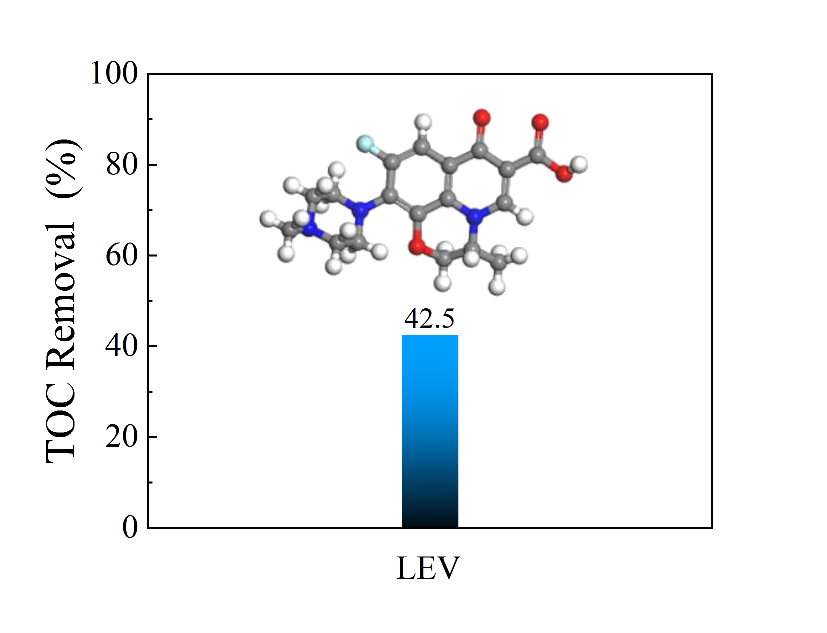
**

Figure S6 TOC removal during the photocatalytic degradation process for LEV (10 mg L^-1^, 50 mL) by AB-9.

Table S1 Possible intermediates for LEV degradation using AB-9 as the catalyst under visible-light irradiation.

| **Substance** | **Formula** | **m/z** | **Potential structure** |
| --- | --- | --- | --- |
| Levofloxacin (LEV) | C_18_H_20_N_3_O_4_F | 362 |  |
| P1 | C_18_H_18_N_3_O_6_F | 392 |  |
| P2 | C_16_H_17_N_3_O_6_ | 348 |  |
| P3 | C_15_H_17_N_3_O_4_ | 304 |  |
| P4 | C_13_H_12_N_2_O_5_ | 277 |  |
| P5 | C_7_H_5_NO_4_ | 168 |  |
| P6 | C_17_H_20_N_3_O_2_F | 318 |  |
| P7 | C_17_H_20_N_3_O_5_F | 366 |  |
| P8 | C_7_H_7_N_2_O_2_F | 171 |  |
| P9 | C_17_H_18_N_3_O_4_F | 348 |  |
| P10 | C_16_H_18_N3O2F | 304 |  |
| P11 | C_14_H_18_N_3_O_2_F | 280 |  |
| P12 | C_10_H_11_N_2_OF | 195 |  |


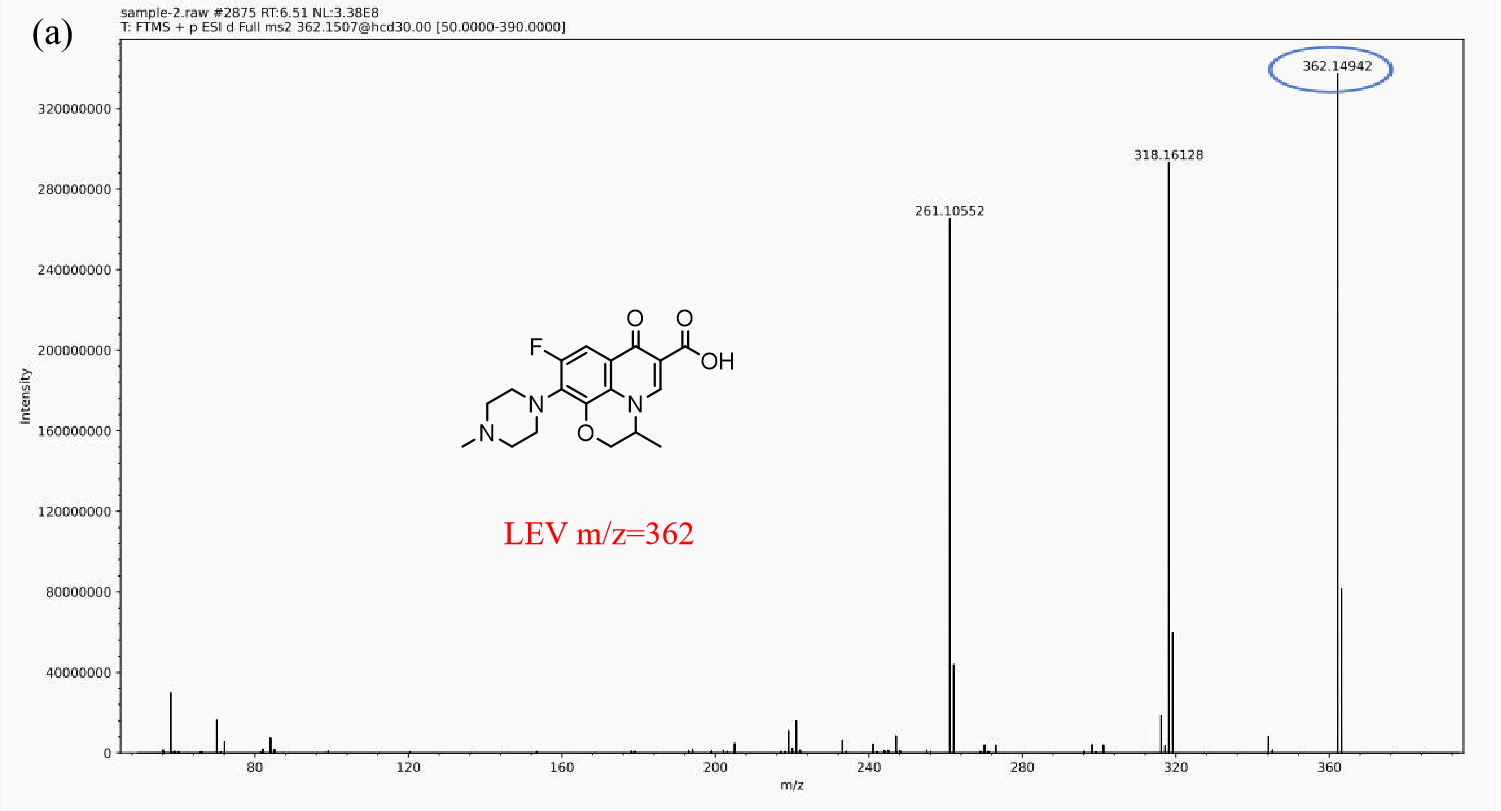


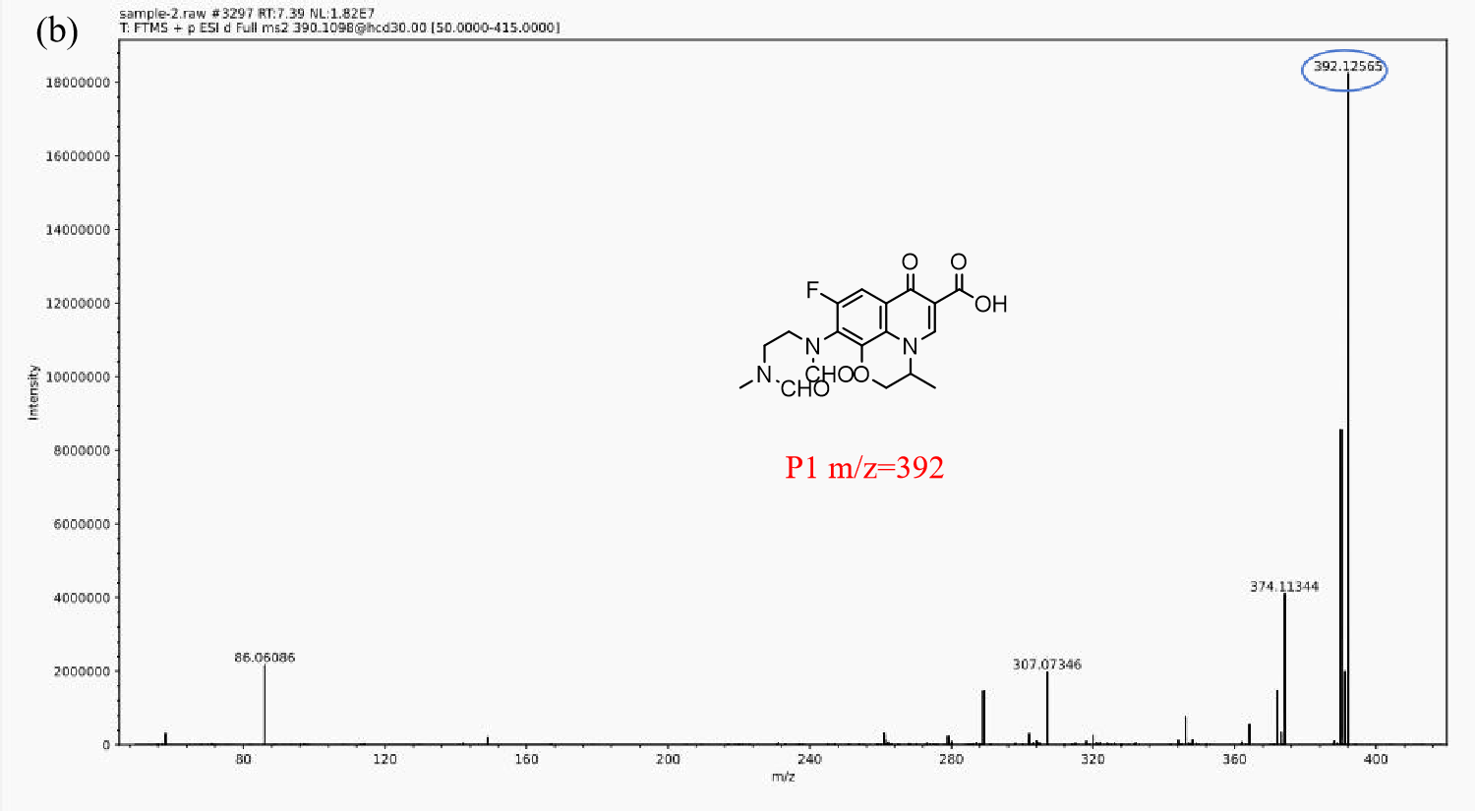


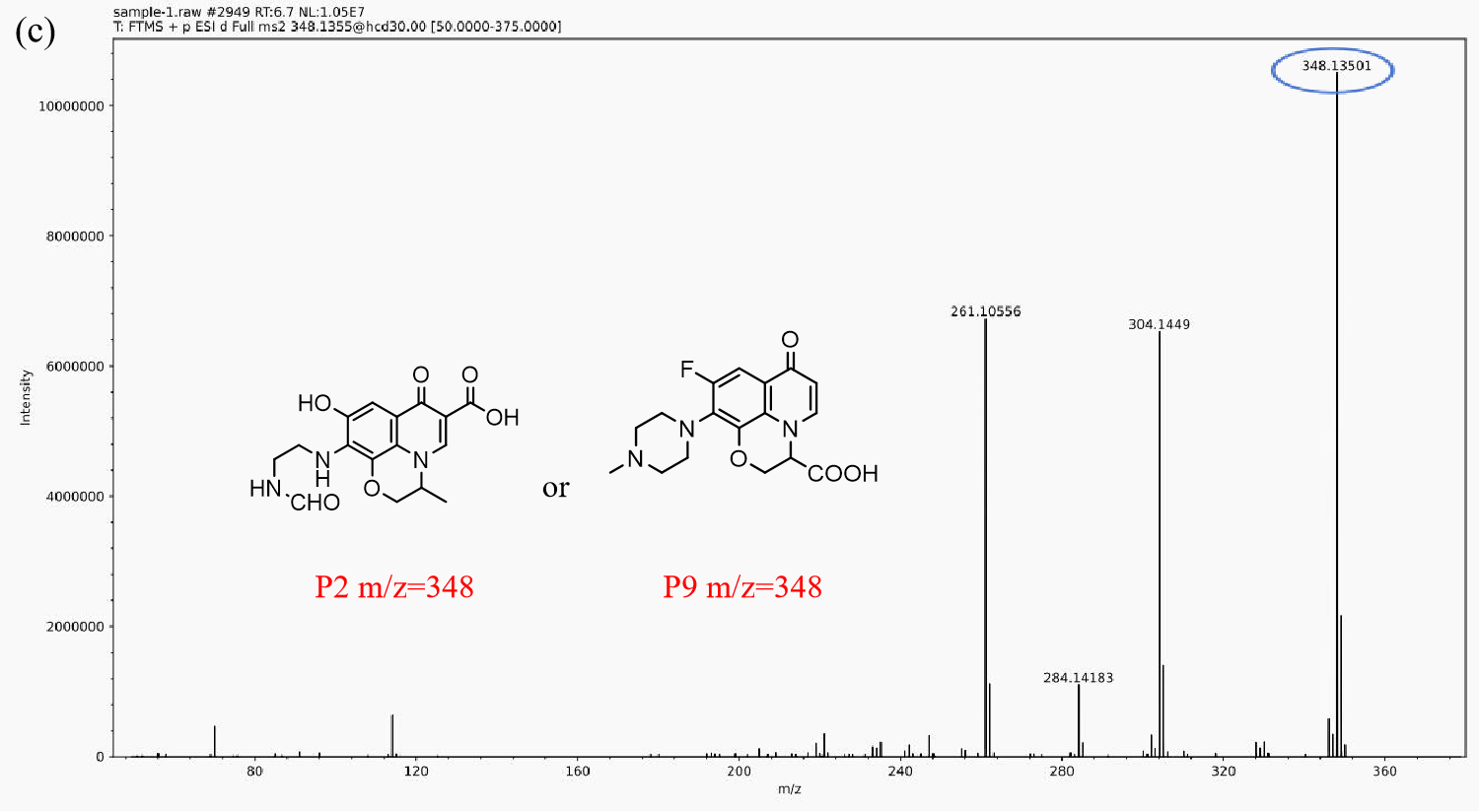


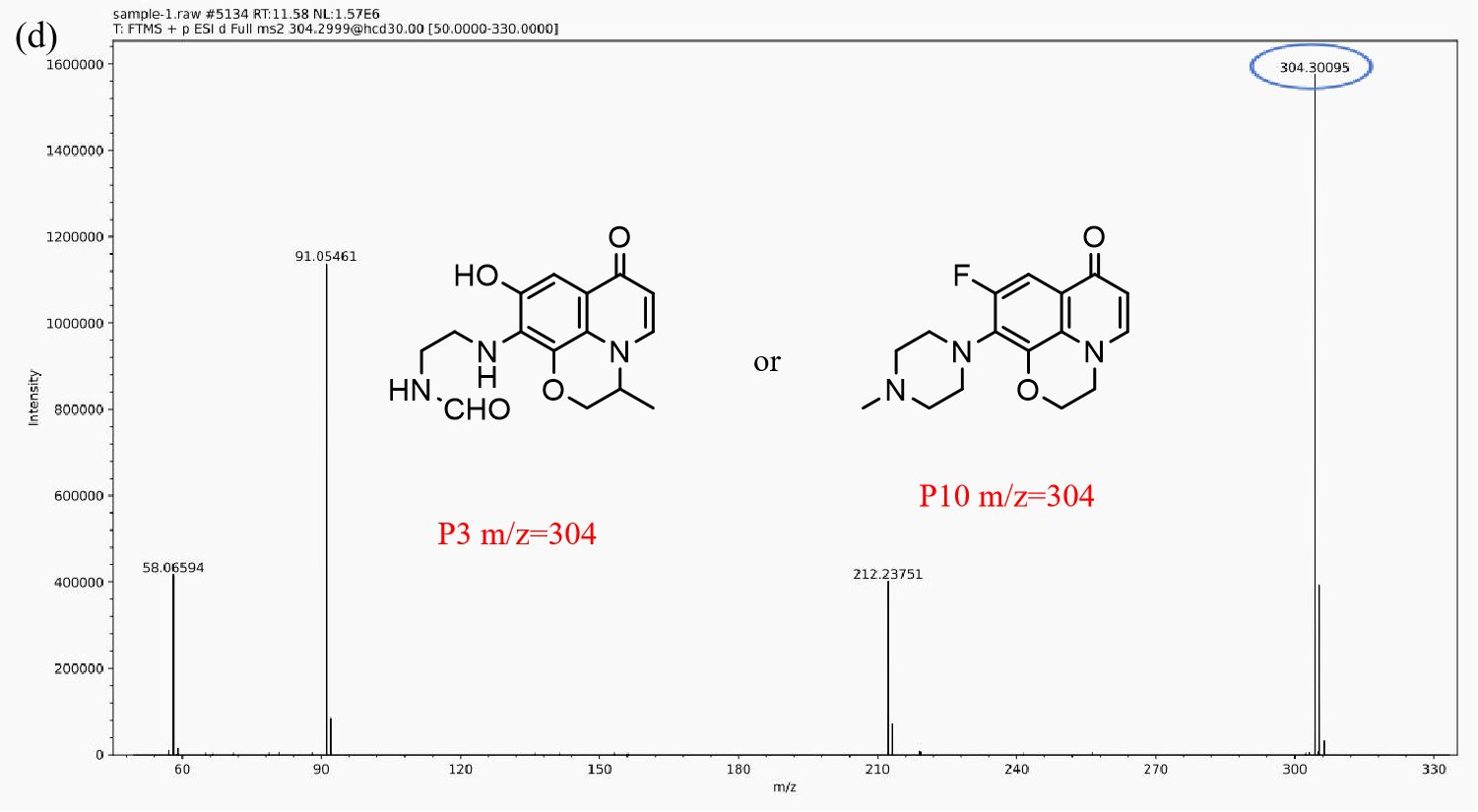


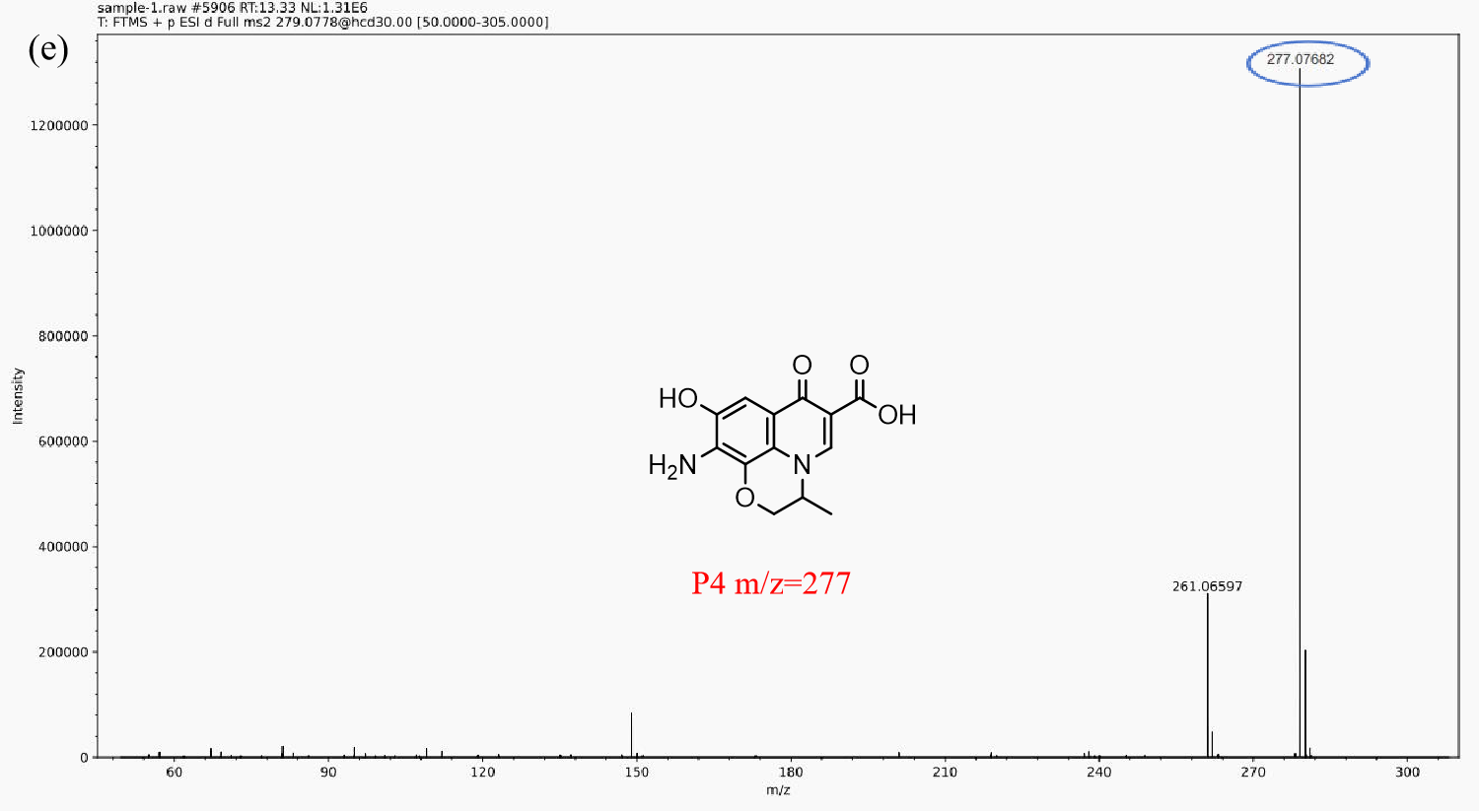


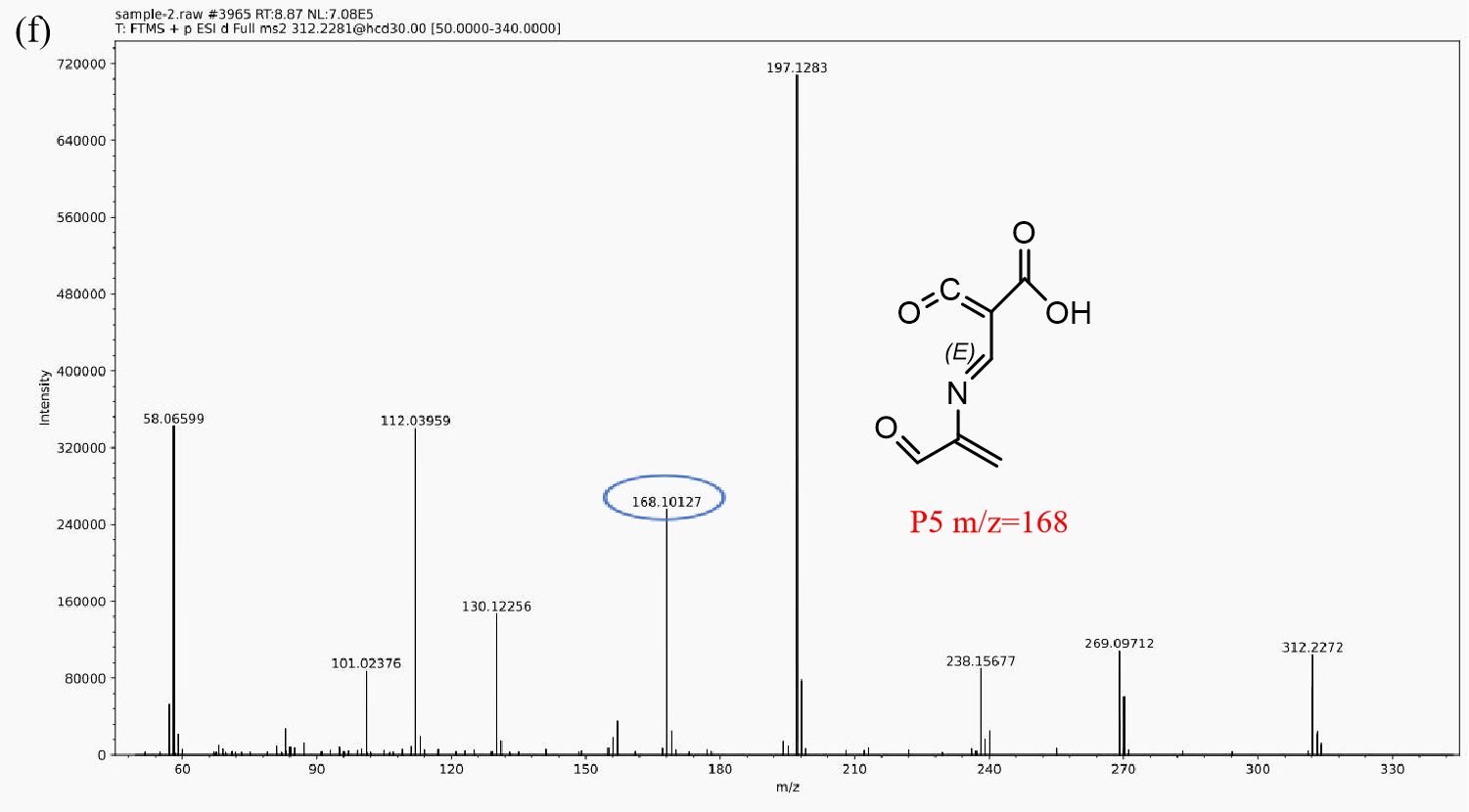


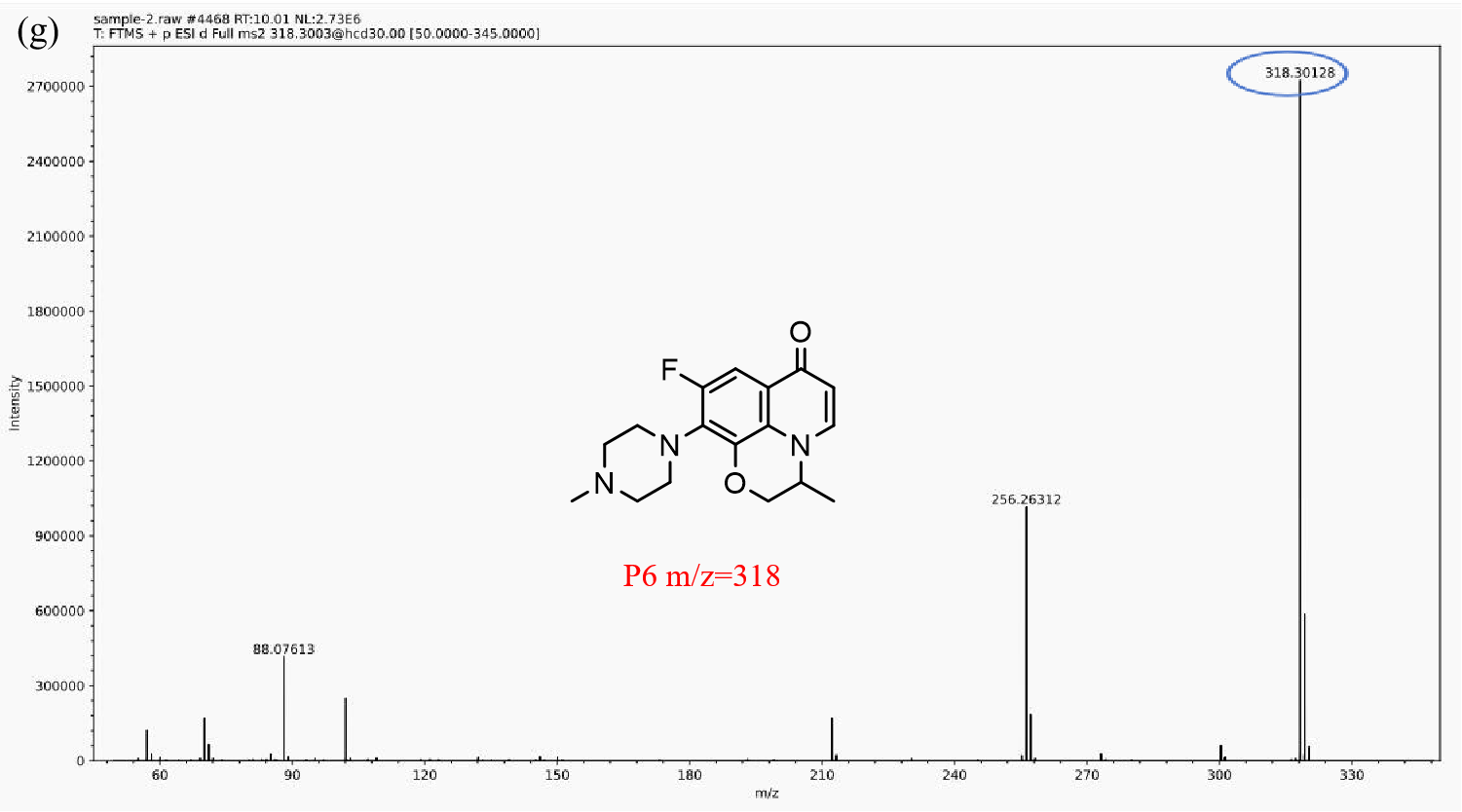


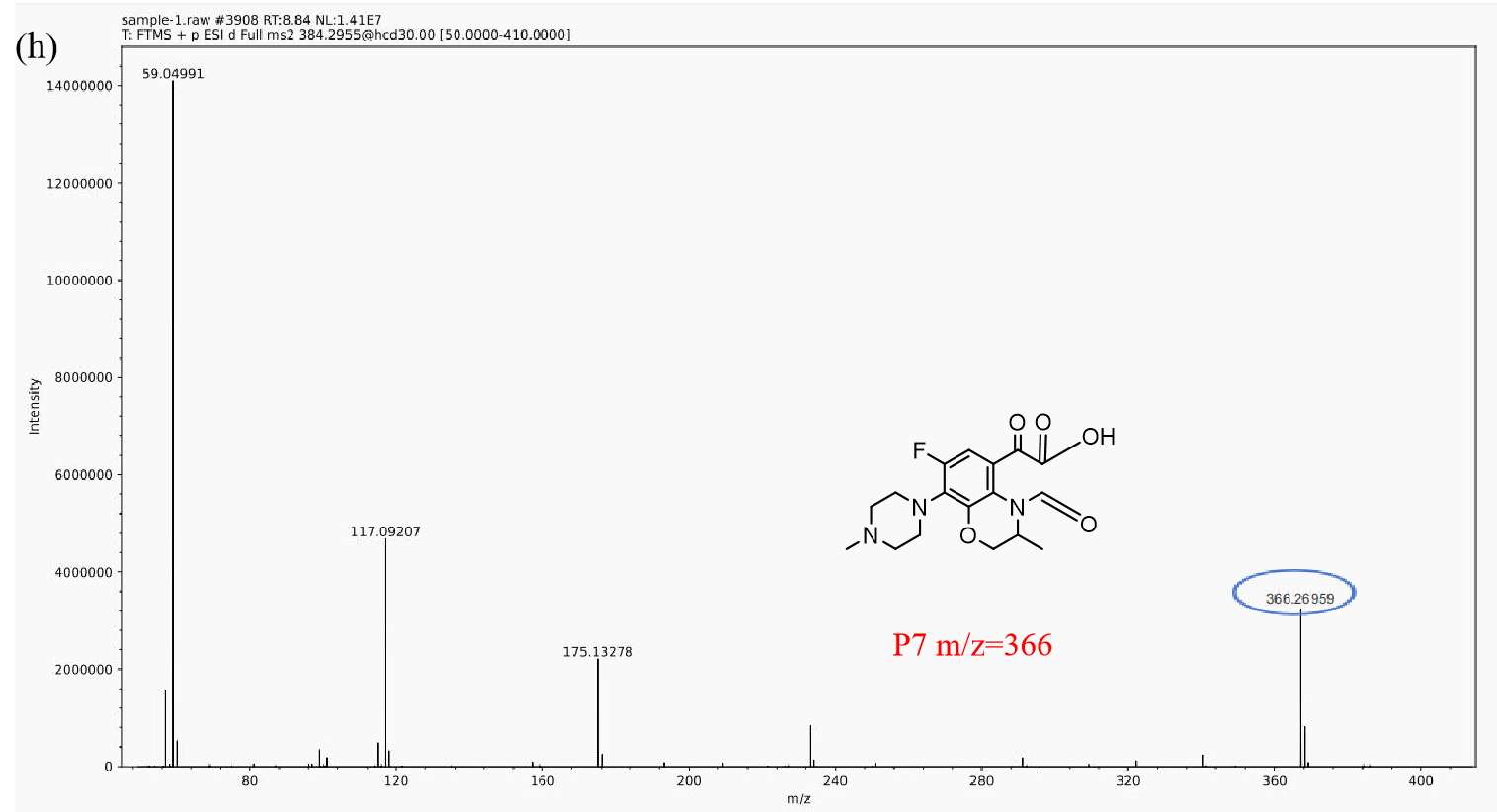


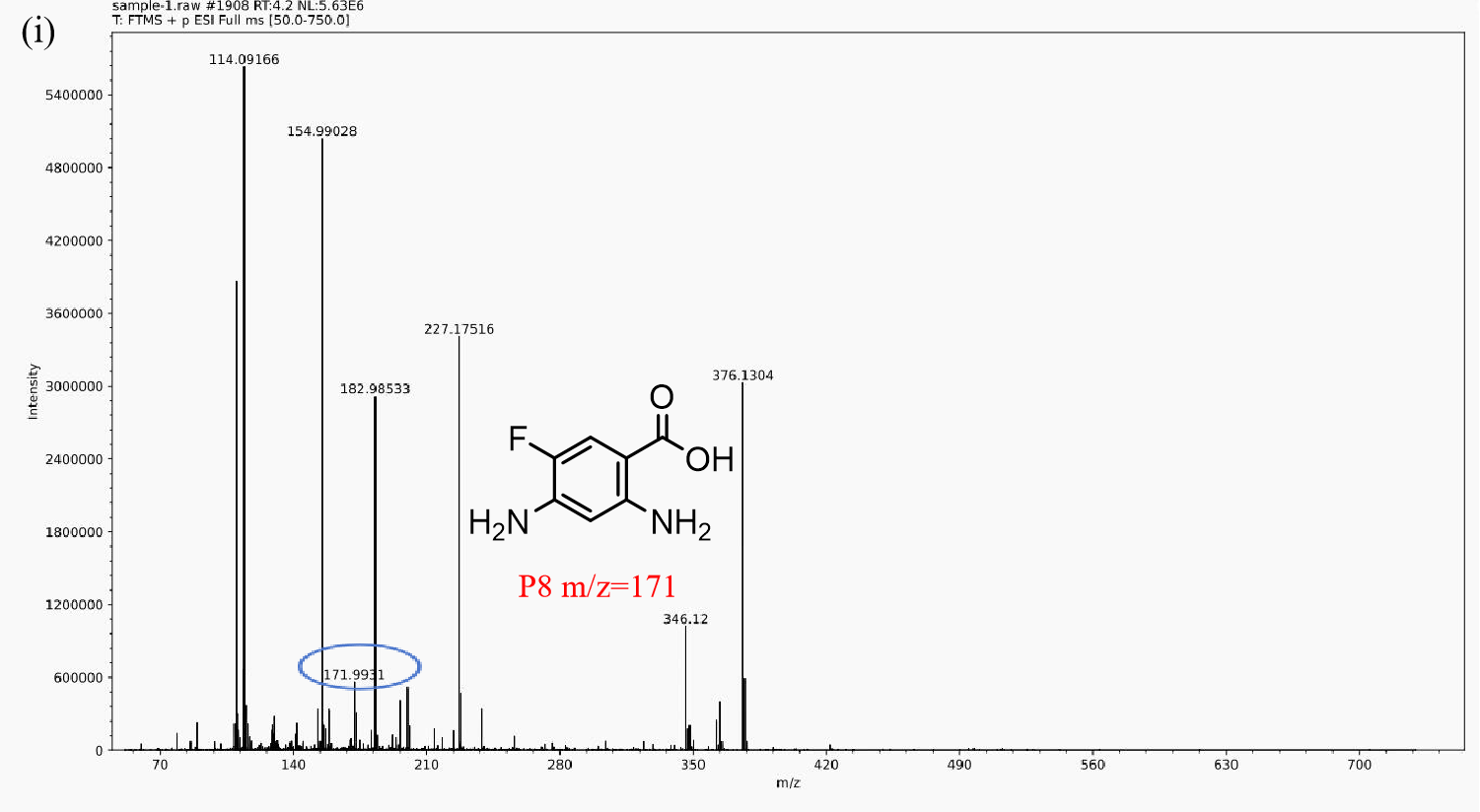


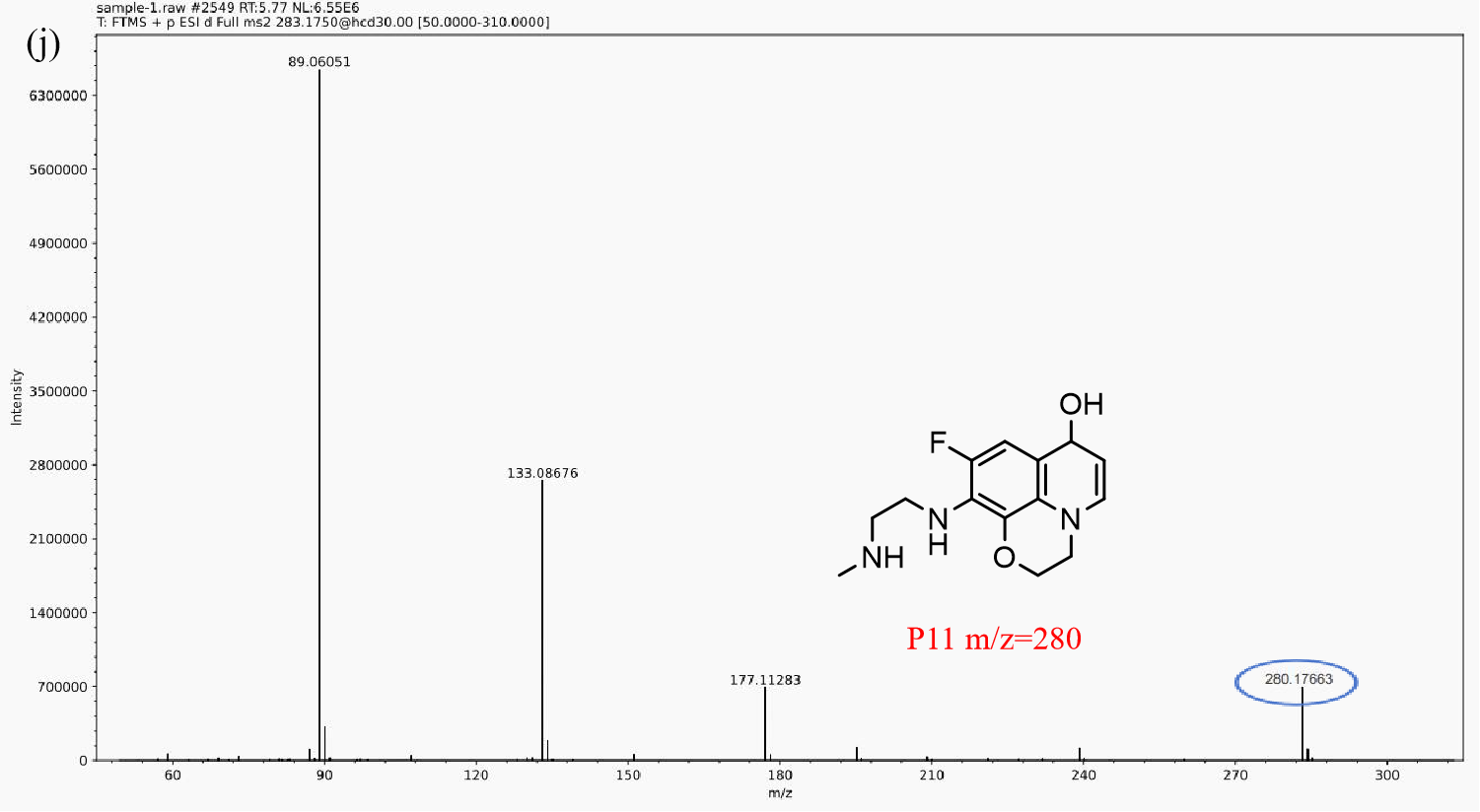


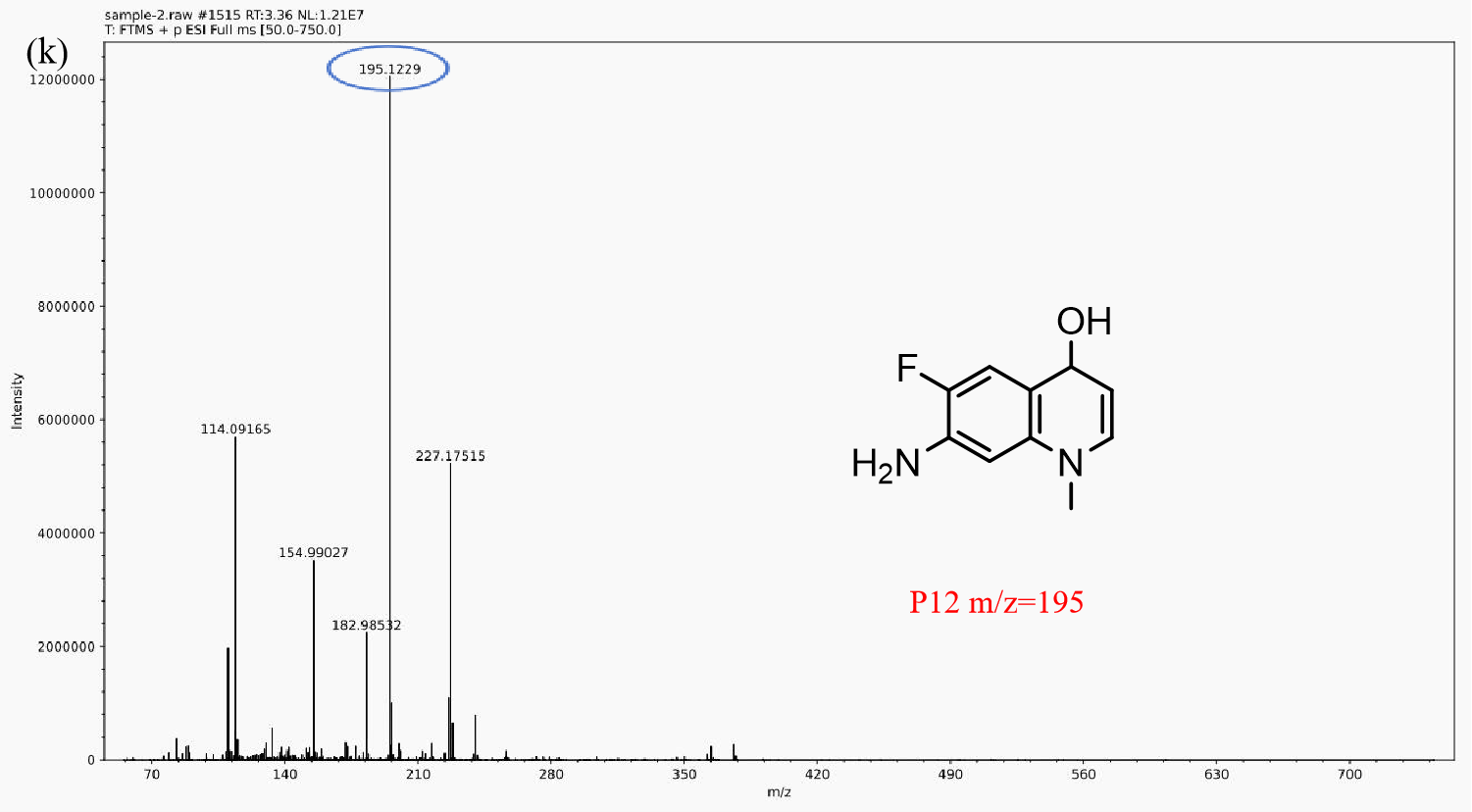


Figure S7 (a-k)LC-MS spectra of [M+H]^+^ ions of LEV and possible intermediate products of LEV after visible light irradiation of 60 min
